# High-performance printed electronics based on inorganic semiconducting nano to chip scale structures

**DOI:** 10.1186/s40580-020-00243-6

**Published:** 2020-10-09

**Authors:** Abhishek Singh Dahiya, Dhayalan Shakthivel, Yogeenth Kumaresan, Ayoub Zumeit, Adamos Christou, Ravinder Dahiya

**Affiliations:** Bendable Electronics and Sensing Technologies (BEST) Group, University of Glasgow, Glasgow, G12 8QQ UK

**Keywords:** Printed Electronics, Large area electronics, Contact printing, Transfer printing, Flexible electronics, Nanomaterials, Nanostructures, Ultra-thin chips, High-Performance

## Abstract

The Printed Electronics (PE) is expected to revolutionise the way electronics will be manufactured in the future. Building on the achievements of the traditional printing industry, and the recent advances in flexible electronics and digital technologies, PE may even substitute the conventional silicon-based electronics if the performance of printed devices and circuits can be at par with silicon-based devices. In this regard, the inorganic semiconducting materials-based approaches have opened new avenues as printed nano (e.g. nanowires (NWs), nanoribbons (NRs) etc.), micro (e.g. microwires (MWs)) and chip (e.g. ultra-thin chips (UTCs)) scale structures from these materials have been shown to have performances at par with silicon-based electronics. This paper reviews the developments related to inorganic semiconducting materials based high-performance large area PE, particularly using the two routes i.e. Contact Printing (CP) and Transfer Printing (TP). The detailed survey of these technologies for large area PE onto various unconventional substrates (e.g. plastic, paper etc.) is presented along with some examples of electronic devices and circuit developed with printed NWs, NRs and UTCs. Finally, we discuss the opportunities offered by PE, and the technical challenges and viable solutions for the integration of inorganic functional materials into large areas, 3D layouts for high throughput, and industrial-scale manufacturing using printing technologies.

## Introduction

**Fig. 1:**
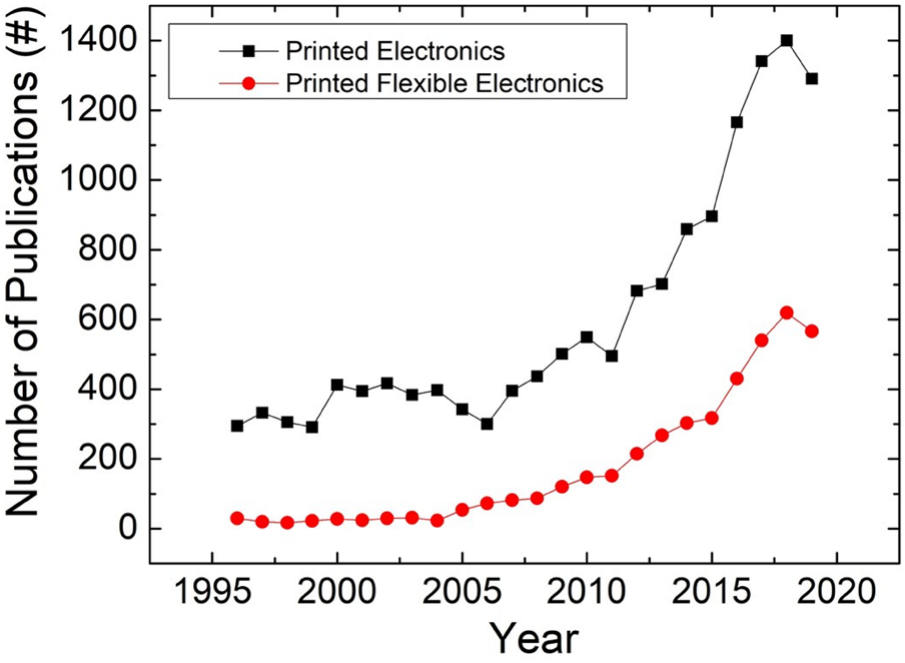
Year-wise number of publications in printed electronics. The data for these plots were taken from the Web of Science by using relevant keywords (e.g., Printed Electronics). The figure also shows number of publications in printed flexible electronics

There is growing interest in developing large-area flexible electronics for applications across numerous sectors, including wearables, robotics, consumer electronics, and healthcare [1–6]. Flexible electronics has several advantages such as conformability to different shapes, which make it indispensable for above application areas where electronic devices are needed on unconventional substrates to either conform to curvy surfaces or to degrade naturally [7–20]. Accordingly, significant research efforts are being made to develop electronic devices and systems with flexible form factors and novel manufacturing technologies [9, 11, 21–30]. These range from integrating off-the-shelf electronic devices on flexible printed circuit boards to printing functional inks and materials to realise active devices and circuits [21, 31]. Among these technologies, the Printed electronics (PE), defined as the printing of circuits on diverse planar and non-planer substrates such as paper, polymers and textiles, has seen rapid development motivated by the promise of low-cost, high volume, high-throughput production of electronic devices [22]. The growth in the number of publications (Fig. 1) related to PE in recent times just indicates this advancement.

**Fig. 2:**
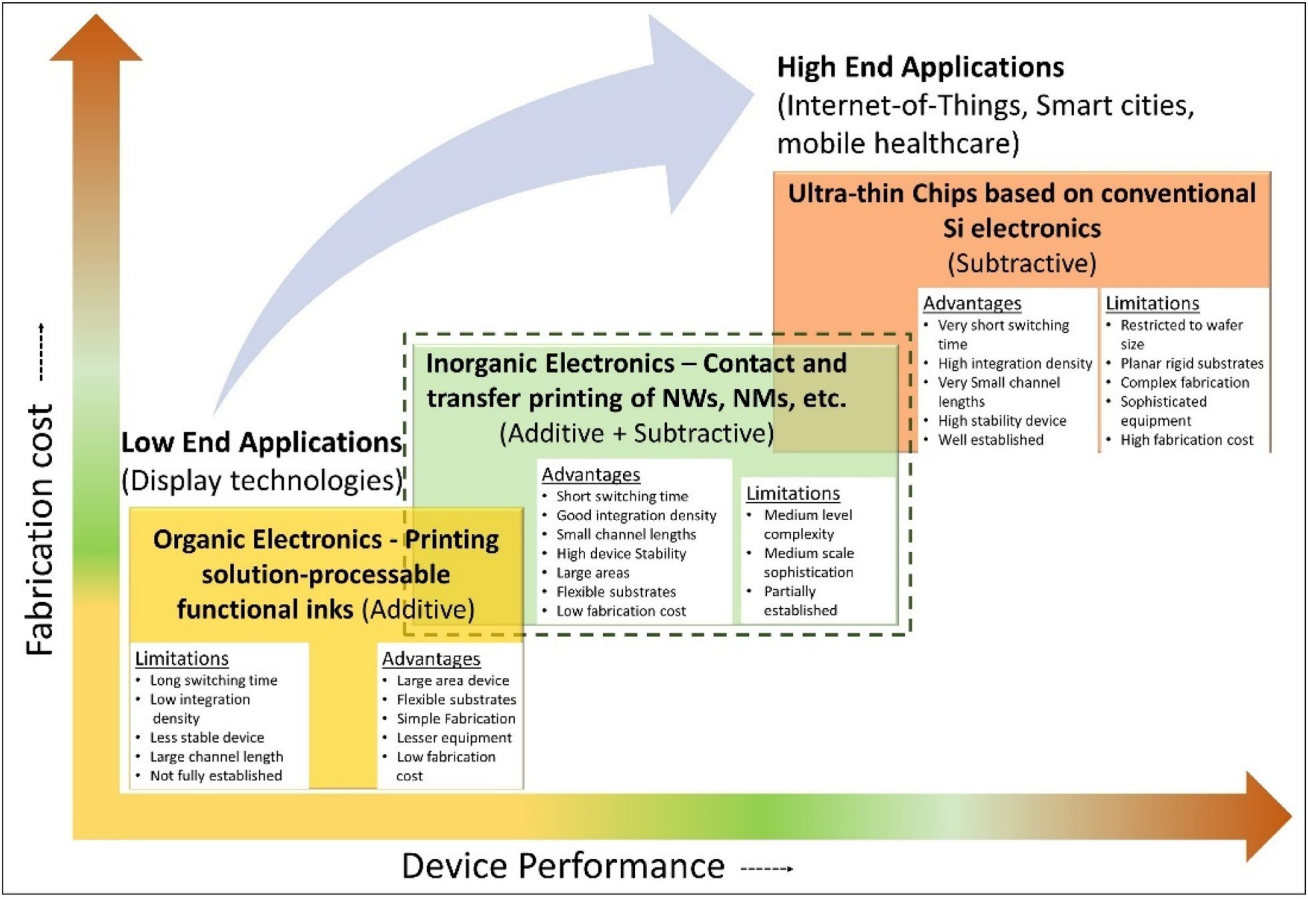
Different manufacturing routes for the fabrication of large area electronics and the relation between fabrication-cost and device-performance. Inorganic printed electronics could potentially provide the long-term solution for high performance large area electronics

For more than five decades, the silicon based conventional electronics has dominated the high-end electronic applications with ever growing integration density and the high transistor switching speed. Currently, the PE is not considered as the substitute for conventional silicon-based electronics. Instead the niche market for PE is considered to lie in the low-cost printed circuits based on conducting, organic semiconducting and dielectric materials based inks aiming at high-volume market segments, where the high performance of conventional electronics is not required [4, 32–35]. This is owing to the modest performance (e.g. slower transistors switching speed) offered by the devices based on printed organic semiconducting materials, which otherwise offer excellent features, including intrinsic flexibility, light weight, and low cost (in general, the cost of PE is expected to be two or three orders of magnitude cheaper than Si per unit area). The chemical instability and poor charge mobility also limit their use to the low-end applications [36, 37]. On other hand, several emerging applications (e.g. Internet of Things (IoT), mobile healthcare (m-healthcare), smart cities, robotics, etc.) require fast computation and communication large-scale integration, for example, to achieve a processing engine [38, 39]. As a result, alternative approaches such as metal-oxide based thin-film transistor technology are being explored, even with more complicated layouts [38]. This is because in the absence of viable p-type material, only n-type field-effect transistors (FETs) can be fabricated. Likewise, metal-oxide based printed sensors have been reported [40, 41]. The single-crystalline inorganic semiconducting materials-based PE is other route that has attracted significant interest recently with innovative printing of nano (e.g. nanowires (NWs), nanoribbons (NRs) or nanomembranes (NMs) etc.), micro (e.g. microwires (MWs)) and chip (e.g. ultra-thin chips (UTCs)) scale structures [25, 26, 42–53]. These nano/micro/chip scale structures are typically single-crystalline and result in high-performance electronics. For instance, the mobility (in cm^2^/V·s) of about 270 for n-type Si-NWs [54], 300 for p-type Si NWs, 730 for Ge/Si core/shell NWs [55] and 660 for Si NRs [46] have been demonstrated. The transfer printing (TP) and contact printing (CP) methods developed to transfer or print these structures onto flexible substrates could address the traditional thermal budget issue associated with the inorganic semiconducting materials i.e. due to high-temperature processing requirements it is difficult to fabricate devices directly over flexible polymeric substrates [26, 29, 47, 49, 56]. Figure 2 shows a qualitative comparison of these technologies in terms of fabrication-cost and device-performance along with their advantages and limitations.

The possible high-performance, at par with silicon-based electronics, with printed inorganic semiconducting materials-based devices has revived the discussion about PE as substitute for the conventional silicon-based electronics [57, 58]. With combined features such as low fabrication cost and high-performance, the inorganic semiconducting materials-based PE could provide a means to implement innovative solutions such as large area ultra-thin electronic skins (eSkin) for ubiquitous computing and pervasive context-sensitive inter-object interaction [21, 31, 59–61]. PE also leads to less materials wastage, which could help to reduce the electronic waste (e-waste) and potentially allow reuse of some of the electronic materials (e.g. conductive inks) to open new avenues towards circular electronics. Considering these developments, it is opportune time to review the latest advances in PE technologies and new opportunities they offer through high-performance devices. Most of the surveys on PE so far have focussed on organic materials-based electronics and the low-end applications from them [62–66]. A few reviews have also focused on inorganic nanomaterial synthesis [61, 67–70], printing technologies [22, 71, 72], transfer printing of either chip scale UTCs [49] or inorganic micro/nanostructures [73] and applications [29, 49, 73]. For high-performance electronics, these works mainly focus on the high mobility of the inorganic materials. While this is an important factor, the technological parameters such as channel lengths and ohmic junctions etc. also influence the performance of devices and require more attention. Considering these factors and the application potential of high-performance PE, this paper provides a thorough review of printing technologies for nano to chip scale inorganic semiconducting structures, mainly in 2D layouts. High-performance soft electronic devices and circuits from assembly of multitude of advanced inorganic materials of various dimensions, including nano (NWs, NRs, NMs etc.), micro (MWs), and the chip scale (UTCs) etc. are presented. Further, we discuss the potential of these techniques for two-dimensional materials (Graphene) and simultaneous printing of multi-materials (heterogeneous integration) in three dimensional (3D) layouts.

This paper is organized into five sections: In section II, we briefly present the synthesis of nano to chip or macro (e.g. wafer) scale inorganic elements including NWs, NRs, NMs and UTCs. Section III describes the CP and TP technologies for printing NWs, NRs, NMs and UTCs etc. In Section IV, we present few examples of printed inorganic materials based flexible electronic devices. We conclude the review in section V, where we summarise the key developments and present an overview of the main challenges for high-performance PE along with potential solutions.

## Printable inorganic nano to macro scale structures: fabrication methods and techniques

**Fig. 3:**
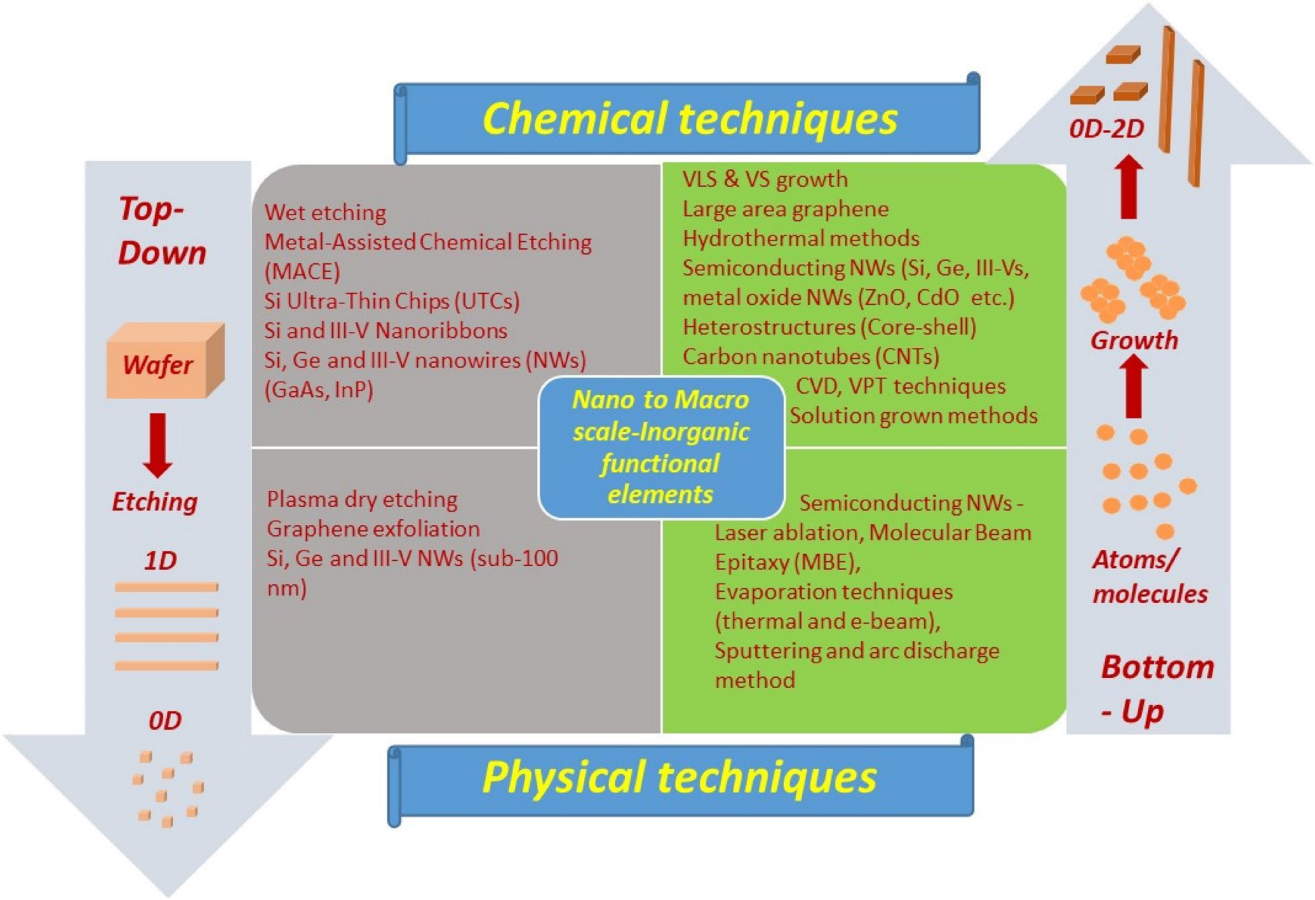
Experimental domain for the fabrication of the various inorganic functional elements. Left column shows the chemical and physical techniques used for top-down approaches and right column represents bottom-up approaches

The inorganic materials-based nano to macro scale structures (sub-100 nm to wafer scale) discussed above could be fabricated using either bottom-up or top down approaches through wide variety of physical and chemical techniques, as summarised in Fig. 3 [70, 74–78]. The developed techniques largely aim to produce structures with precisely controlled dimensions and chemical composition which are crucial for the development of novel flexible devices (e.g. FETs [79, 80], light-emitting diodes [81], thermoelectric [82] and piezoelectric nanogenerators [83–85] etc.). The combination of bottom-up or top-down strategy and the experimental technique are decided based upon the material used and the application requirement. PE technologies bridge these two (materials fabrication and applications) fronts by creating ensemble of aligned nanostructures over flexible substrates. Innovative methodologies have been developed for printing materials or structures with dimensions ranging from few nm’s to chip scale to create single functional units. The orientation (i.e. vertical or horizontal) of the inorganic materials over the source wafers or substrate is determined by the fabrication process and eventually influences the printing technique. For example, lithographic and plasma dry etching-based approaches normally produce horizontal array of Si micro/nano ribbons which are suitable for TP. Differently, bottom up NWs with diameters in the range of few nm to several 100 s of nm, commonly grown vertically over rigid substrates, are suitable for CP.

### Nanoscale structures

Various top-down and bottom-up approaches, have allowed large area synthesis of III–V NWs, metal oxide NWs and IV NWs at wafer scale [73]. This section briefly describes the fabrication and growth of inorganic NWs under different conditions (dry and wet etching, high temperature ambient etc.) in the dimensional range from few nm’s to areal coverage larger than wafers.

#### Top-down approach

**Fig. 4:**
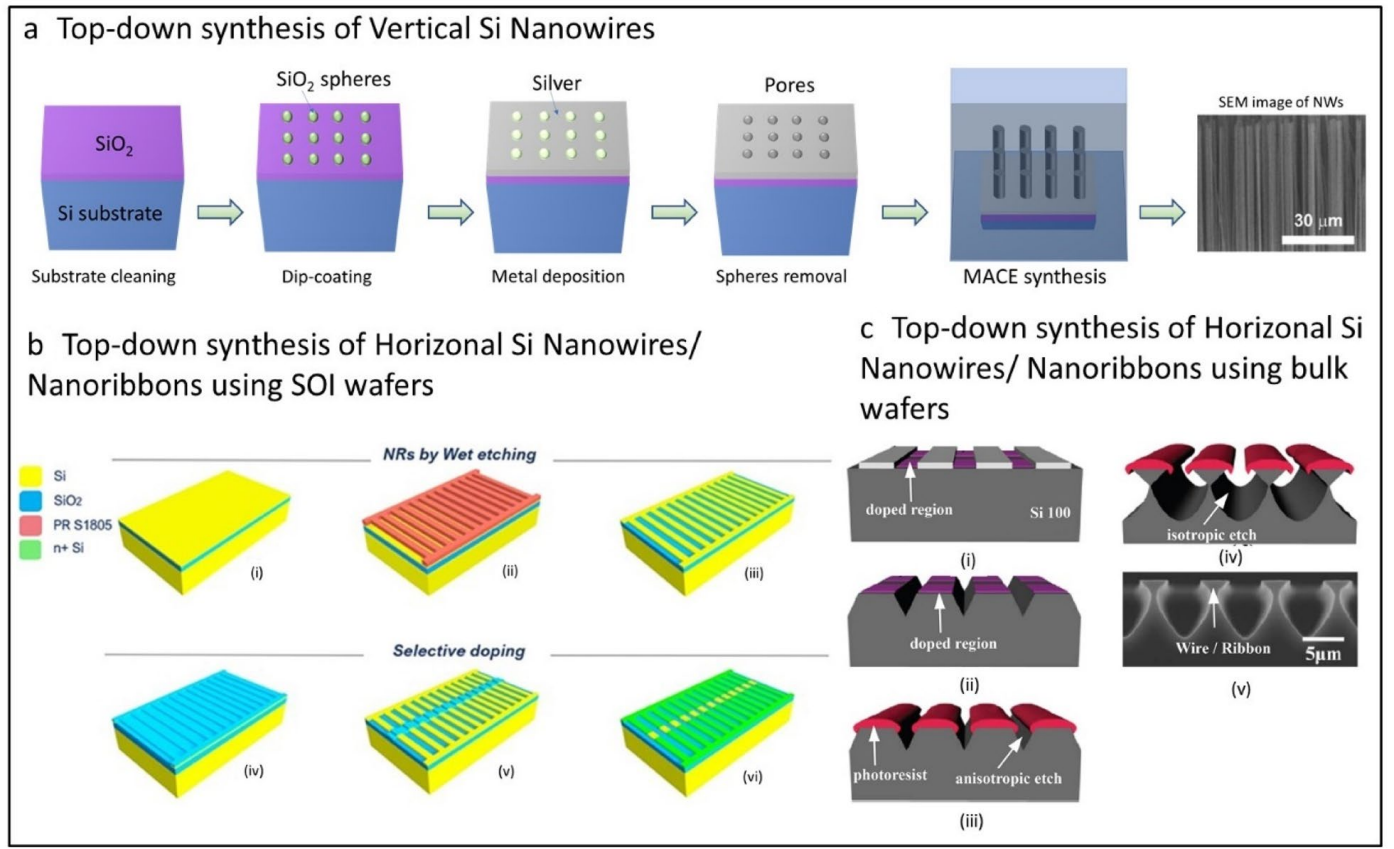
Schematic representation of the growth of nano scale structures via top-down methods. **a** Silicon nanowires using metal assisted chemical etching (MACE) process. Schematics adapted from [59]. **b** Schematic diagram illustrates stages of the Si NRs fabrication and selective doping (i) The source wafer consists a layer of active Si < 100 > with 70 nm thick, on top of 2 µm of BOX, supported by 600 µm bulk Si. (ii) Si NR’s geometry is defined by conventional UV lithography procedure. UV lithography is performed by spin coating photoresist, followed by soft baking, the samples are exposed to UV source and subsequentially the NRs definition are developed. (iii) Dry etching is performed in this step by reactive ion etching (RIE) using a combination of CH_3_/O_2_ gas sources, to finalize the structure of NRs structure after photoresist removal using acetone and IPA. (iv) The first step to perform a selective n + type doping of active regions (source/drain for FETs) is by applying plasma enhanced chemical vapour deposition (PECVD) of SiOx layer. (v) The SiOx barriers over the active regions are etched away by dry etching. (vi) Spin on dopant method of phosphorus is used to create ohmic contacts at source and drain regions on the source wafer while the channel is masked by SiOx, served as a barrier. A wet etching method using hydrofluoric acid is performed to remove both dopant diffusion barrier layer and buried oxide layer leading in release of the NRs from the bulk wafer, allowing the selectively doped NRs to be transfer printed to any flexible substrate. Adapted with permission from [46]. **c** Schematic illustration of the fabrication of NWs/NMs by use of anisotropic wet-chemical etching techniques applied to bulk wafers

Top down approach is commonly carried out by selective etching of single crystalline wafers using wet chemicals or plasma processes. The process starts with a wafer such as Si, Ge, GaAs, which determine the final crystallinity and chemical composition of the structures (NWs, NMs, NRs etc.) to be printed. The NWs are produced by employing nano-patterning techniques such as lithography (optical and e-beam) [25, 86, 87], nanosphere based patterning [88, 89], laser interference lithography [90, 91], etc. The etching could be done using dry plasma process [92, 93] or acid based wet chemical etching [25, 86, 87, 91]. Dry etching methods have merits such as high precision, uniformity over wafer scale, scalability etc., but they could also lead to stress generation due to high energy plasma and isotropic lateral etching issues. Alternatively, HF acid-based metal assisted chemical etching (MACE) (Fig. 4a) of Si is the most cost-effective technique to date for the fabrication of sub-100 nm Si nanostructures [88, 89]. The selective etching of Si wafer could be carried out using patterns produced with nanosphere lithography (Fig. 4a). These NWs could be printed on various substrates using CP technique to obtain the nanoscale electronic layers, which are eventually used for devices. Using top-down means, synthesis of semiconducting NWs of different materials such as Si [25, 88], InP [94], GaN [95], GaAs [86, 87, 96] etc. have been demonstrated.

Top-down methods have also been used to obtain horizontally aligned NRs/NMs from SOI wafer with thickness ranging from few ‘nms' to few tens of nm and lateral dimensions between few tens of µm to mm. Their fabrication process involves anisotropic wet or dry etching of selected exposed regions on the top side of Si wafer, and then undercut removal of the buried oxide (BOX) with hydrofluoric acid to release Si NRs or NMs structures [46, 97–100]. Figure 4b illustrates one such example where the optical lithography and wet chemical etching steps are followed to obtain Si NRs from commercial SOI wafer [46]. This technique produces horizontal array of NRs over SOI source wafers and these are eventually transfer printed over flexible substrates. Figure 4b also illustrates the steps for achieving ohmic contacts on NRs/NMs by selective doping which is critical for achieving high device performance (discussed in later sections). The top-down approach has also been used to develop NWs/NRs from bulk wafers (Fig. 4c) [101–103], however, the dimensional control with SOI wafer is much better. Many compound semiconducting materials including GaAs [104–107], GaN [108], and InP [109] have been obtained in a conceptually similar manner to that of Si shown in Fig. 4b–c. The major limitation of the top-down approach is the requirement of single crystalline wafers, which is not available for many technologically important III-V and oxide semiconductors.

#### Bottom-up approach

**Fig. 5:**
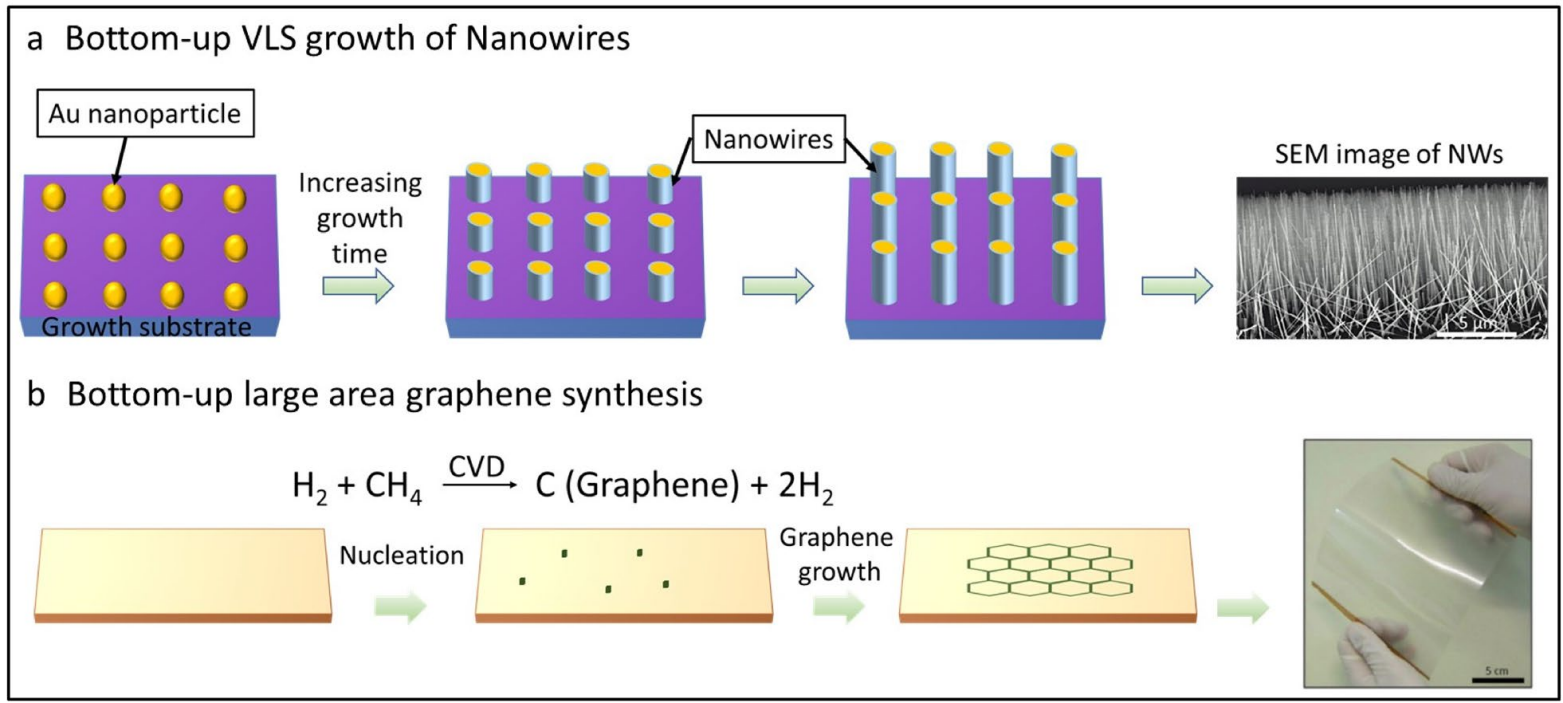
Schematic representation of the growth of nano scale structures via bottom-up methods: (**a**) VLS growth of nanowires and (**b**) CVD based large area graphene synthesis [122]

The bottom-up approaches have been widely used to grow single crystalline 1D materials using atomic and molecular precursors exploiting well known physical and chemical techniques (chemical vapour deposition (CVD), vapour phase transport (VPT), supersaturated solutions etc.). The major advantage of the bottom-up approaches is their ability to precisely tune crystallinity and composition during the growth process. Synthesis strategies, including catalyst particle assisted vapour-liquid–solid (VLS) [75, 110–112], vapour–solid (VS) [113–116], vapour–solid–solid (VSS) [117–121], and low-temperature solution-based processes such as hydrothermal [74, 79, 83] have been widely exploited for growing inorganic NWs. The VLS growth of NWs (Fig. 5a) is one of notable method to produce single crystalline and composition controlled semiconducting NWs in the sub-100 nm regime. The VLS mechanism offers many advantages such as single crystallinity, in situ composition control, site specific growth, precise dimensional control in sub-100 nm regime and ability to produce heterostructures (core–shell and axial) with sharp interfaces. These wide process variability offers many advantages for the development of high-performance PE. The VLS growth process produces vertically oriented high aspect ratio NWs over rigid substrates which are compatible for contact and roll-to-roll (R2R) printing [59]. Bottom up method have been successfully used for 2D nanomaterials which are key components for flexible large area electronics. For example, the growth of monolayer (ML) graphene over Cu substrate under CVD conditions (Fig. 5b) (temperature over ~ 1000 °C and methane (CH_4_) source) has been demonstrated over areas ranging from few mm to several centimetres [122]. The large area graphene has been seamlessly transferred over flexible PVC substrates using solution-based transfer process (Fig. 5b). Bottom up approaches largely use high temperature (> 600 °C) processing conditions to produce high quality inorganic materials needed for high-performance flexible substrates. Various printing technologies bridging the high temperature growth processes with low temperature device fabrication steps are discussed later in Sect. 3.

### Microscale structures

Just like the NMs and NRs, the microscale structures have been successfully fabricated from SOI wafers using top-down methods, as described in Sect. 2.1.1. The microstructures of different materials such as Si microwires [99, 100, 123], GaN microwires [108], lead zirconate titanate (PZT) ribbons [124], GaAs and GaN microwires [87, 108] etc. have been demonstrated in the literature. The examples of high-performance electronic devices/circuits using printed microscale structures are discussed later in the Sect. 4.

### Chip scale structures—ultra-thin chips

The chip scale or macroscale structures can be anything with dimensions > 100 μm and can be clearly seen without any microscopy imaging tools. The UTCs are typical example of macrostructures that are obtained by physical or chemical removal of bulk silicon through top-down approaches [49]. Various methodologies used for obtaining UTCs include grinding, controlled spalling technique (CST), dry etching, and wet etching [26, 49, 125–131]. Briefly, the CST is a slim cut process in which thin silicon layer is removed or exfoliated from the bulk silicon chip; the tensile Ni stressor layer is deposited on bulk chip and shear force is applied to generate stress induced parallel fracture along the surface of bulk chip [127]. The UTCs obtained through CST process suffer from deterioration in device carrier concentration and mobility due to the stress induced during CST process [126]. In addition, it is challenging to completely remove the stressor layer. Another popular and broadly utilized thinning approach is back grinding technique, in which the grinding wheel is used to physically dislodge the back/bottom side of silicon to reduce the thickness of bulk silicon down to less than 10 μm with in few minutes [130, 132]. However, the stress induced during the grinding process could alter the silicon crystalline structure resulting in undesirable warping. Alternatively, dry etching process is used to physically dislodge silicon atoms from bulk chip through high energy reactive ions, which is proven to be a stress relief technique to reduce the surface damage [128]. In this case, the high cost, low throughput, and practical difficulties are critical issues. To overcome the cost issue, wet etching process using either tetramethyl ammonium hydroxide (TMAH) or potassium hydroxide (KOH)-based etchant have been widely adopted [26, 125, 133]. The concentration of the etchant and the etching temperature plays an important role in controlling the rate of etching [130]. A detailed discussion and comparison of various UTC technologies is given elsewhere [49]. The transfer process of UTCs will be presented in the next section.

## Technologies for printing of inorganic semiconductors-based nano to macro scale structures

This section describes the two main technologies which have been explored for printing of inorganic semiconductors-based nano to macro scale structures to obtain high-performance PE. These are contact printing (CP) and transfer printing (TP).

### Contact printing

**Fig. 6:**
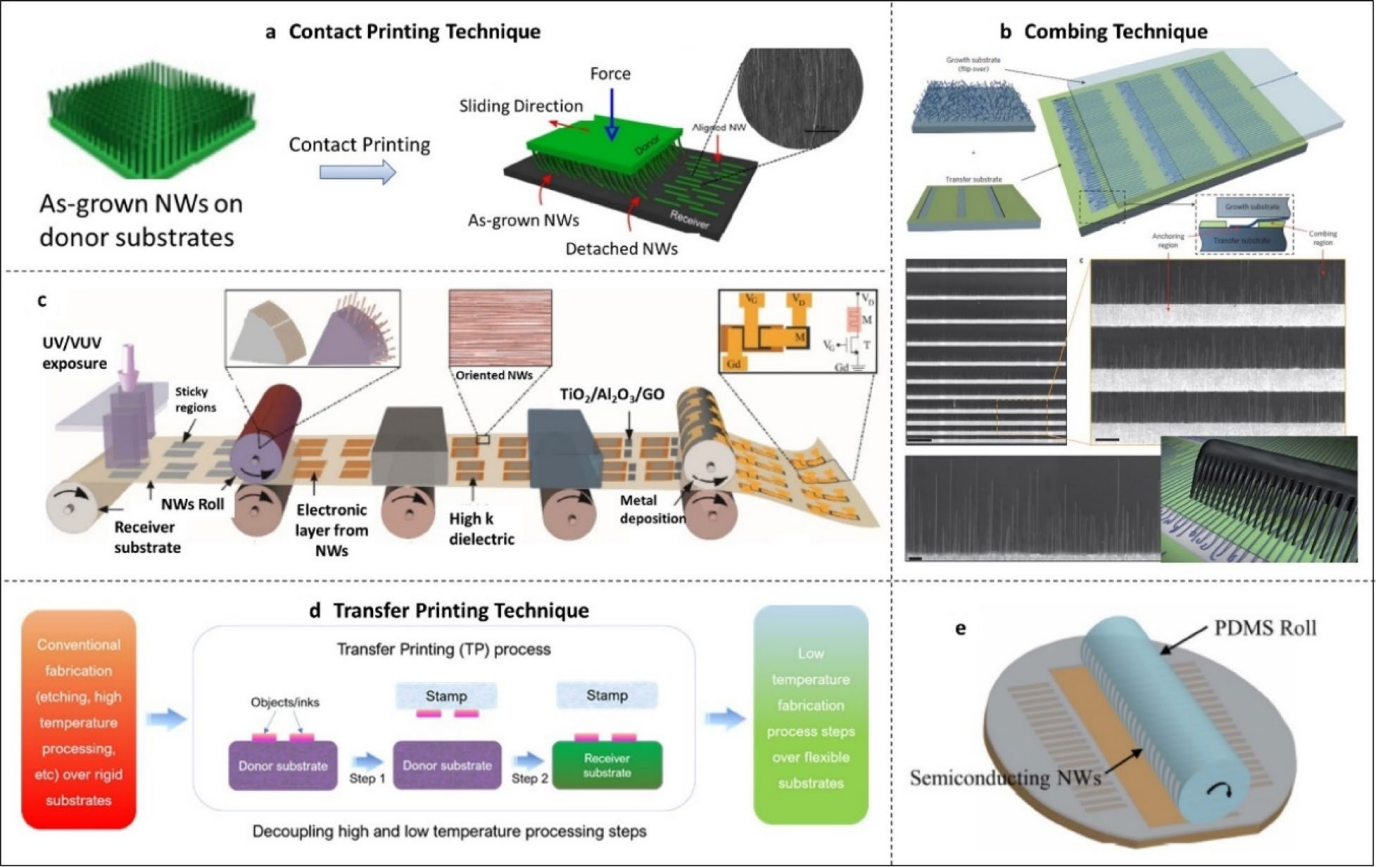
Schematic illustration for: (**a**) the mechanism of contact printing [25], (**b**) combing mechanism [138], (**c**) vision of an R2R production for NW-based functional circuits on large-area flexible electronics [59], (**d**) mechanism for transfer printing [46]. The figure also shows the overview of transfer printing approach enabling transition from high to room temperature processing steps. **e** Concept of roll transfer printing technology

In the CP technique, the nanostructures (usually NWs) come in direct contact with the receiver substrate [22, 25, 42]. Generally, CP is a dry transfer technique which is suitable for printing of any type of bottom-up and/or top-down synthesised vertical NWs. However, modified CP methods have also been developed with controlled surface functionalization (e.g., with −NH_2_ and −N(Me)_3_^+^ terminated monolayers) and/or patterning of the receiver substrate [44, 134]. This method shows excellent density and alignment control of printed NWs, demonstrating the great potential of CP approach for large-area manufacturing in a R2R manner [59], as shown in Fig. 6.

In CP, the as-grown NWs, on their respective donor substrates, are brought in physical contact with the flexible receiver substrate. Then, the donor substrate with vertically grown NWs is pressed against the receiver and then their sliding along a direction leads to transfer of nanostructures (Fig. 6a). The advantage of CP is that the as-printed NWs are transferred onto the receiver substrate in an aligned manner, dictated by the direction of sliding, both on the rigid and flexible substrates. Such alignment is enabled by the shearing force generated during sliding and is favourable for the fabrication of large area device array with uniform high-performance. Specifically, improved NW alignment reduces the variation in NW density across the substrate as well as overlapping of adjacent NWs. The usual ‘grow-harvest-suspend’ [135, 136] route for transferring NWs is not compatible with CP, as the conventional approaches (electric-field-directed assembly, bubble-blown techniques, Langmuir–Blodgett etc.) suffer from poor scalability (about mm-scale), low density, and non-uniformity. The CP allows transfer and alignment in a single step, resulting in a simple process and allowing the use of donors with non-aligned NWs. The method can also be used for printing of NRs and potentially for small (mm scale) flexible chips also [46].

It is important to understand the mechanism of CP to control the printing-process and obtain desired results such as NW density and alignment. The CP has three main steps: (i) NW bending; (ii) alignment of NWs by the applied shear force; and (iii) breakage and transfer of NWs upon anchoring to the receiver substrate through surface chemical and physical interactions [25, 137], as shown in Fig. 6a. The studies related to printing mechanism of NWs as a function of the NW aspect ratio, NW material and applied contact pressure have helped to find the safe range of contact pressures (i.e. without reaching the fracture limit of a single NW) for printing while preserving the maximum original length of NWs [25]. Using COMSOL Multiphysics, these studies have identified several of the key parameters responsible for efficient transfer of NWs, including: (i) the analysis of the maximum strain (ε_max_) and maximum stress (σ_max_) regions along the NW body, (ii) the dependence of ε_max_ and σ_max_ with respect to the NW deflection (δ) and (iii) the dependence of δ with respect to the NW diameter (D) etc. This in-depth study also revealed that the contact printing mechanism requires a continuous and progressive bending of the NWs to reach the fracture strain close to the root of the NW. This can be achieved by using both a constant contact pressure between the donor and receiver substrates and a micrometric sliding stroke.

In practice, the use of NW of different materials and sizes requires extensive optimisation following the simulation analysis. Equipment which can carry out the CP process for different types of NW donor substrates, must offer precise control over the printing parameters [25]. Optimisation is also required in relation to the receiver substrates, with softer flexible martials being damaged due to the large lateral and shear forces applied to detach the NWs from the growth substrate. When considering large area printing, uniformity in the printed array is key as it directly influences the performance variation across devices. Donor and receiver substrate alignment plays a big role in achieving a uniform print [25]. With most NWs being synthesized on flat rigid substrates, optimal plane-to-plane alignment is challenging and R2R approaches could provide better results.

The NW CP technique has been advanced recently with addition of a new alignment method which is called as “combing” [138, 139]. In this process, NWs are anchored to defined areas of a substrate and then drawn out over chemically distinct regions of the substrate (Fig. 6b). This technique has two coexisting steps: the NW anchoring and the NW directional alignment, which are also observed in the conventional CP. However, the combing technique allows the observation and control of both processes individually. While the anchoring force is necessary during the NW printing process, it can dramatically hinder the directional alignment [138, 139]. As an example, the high crossing defect density and the difficulty in realizing the precise registration and position of single NWs in predefined positions is mainly associated with the use of excessive anchoring forces [42]. The combing method has demonstrated good potential to overcome the drawbacks of CP, exhibiting the successful reduction of the crossing defect density down to 0.04 NWs/μm by tuning both anchoring and combing forces. In terms of the realization of a single NW device, the combing method shows great advantages over traditional CP. By controlling the predefined anchor window, the success rate for the realization of a single NW device is ~ 90%: significantly higher than the success rate (~ 60%) achieved using conventional CP [42]. It should also be noticed that while the combing method gives a higher control of NW alignment (~ 98.5% of the NWs aligned to within 1% of the combing direction), the resultant NW density is not comparable to the conventional CP process (~ 9 NWs/μm) [42]. Consequently, the combing method shows more potential in the realization of single NW-based devices. On the other hand, the conventional CP method could be used to achieve high NW density over large area with better alignment. Using CP, modified CP, and combing techniques, printed devices, circuits, and systems based on NWs of high mobility materials have been explored. These examples are discussed in the Sect. 4.

The CP method also shows good potential for roll-to-roll (R2R) manufacturing. For example, rolls of NWs (i.e. NWs on cylindrical substrates) could be used for printing electronic layers as defined locations, as exemplified through Fig. 6c. Alternately, the NWs could be on planar donor substrate and the receiver substrate could be around the cylindrical rolls. The synthesis of NWs on tubes of glass, quartz, and stainless-steel using bottom-up has been demonstrated in the past [162]. By using such rolls in differential roll-printing [162] and roll transfer-printing [163] settings, the contact printing approach can be extended to an R2R-type printing. The example of a R2R process for IT-1 M structures, shown in Fig. 6c, could be the building block for neuromorphic architectures [59, 140].

### Transfer printing

The TP technique enable the transfer of laterally aligned structures or inks from a donor substrate to a receiver substrate generally using soft elastomeric stamp. TP provides a promising solution to fabricate bendable electronic devices at large scale using semiconducting NWs, NRs and UTCs [26, 46, 96, 141].

#### Transfer printing of nano and micro scale structures

The TP technology was primarily explored to overcome the manufacturing problems (e.g. thermal budget issues) associated with the use of traditional microfabrication process for flexible electronics. The concept and mechanism of TP is explained using Fig. 6d. The processing steps that require high temperatures are first carried out on Si wafer (Fig. 4b–c), which can withstand high temperatures and then micro/nanostructures (NWs, NRs, NMs etc.) are picked (step 1) and transferred to flexible receiver substrates (step 2), where further low-temperature fabrication steps are carried out. The mechanism of transfer printing can be understood by studying the competing fracture between the stamp/object interface and the object/substrate interface [29, 142]. During the first step (step 1, Fig. 6d), i.e. object retrieval from the donor substrate, the stamp/object interface must be stronger than the object/substrate interface. On the other hand, to print the object over donor substrate (step 2, Fig. 6d), the stamp/object interface must be weaker than the object/substrate interface. For large area electronics, the controllable and reproducible transfer of micro/nanostructures from the donor to the receiver substrate is needed, and hence the precise control of the interface property is necessary. It is generally assumed that the adhesion strength at the micro/nanostructures—substrate interface is not influenced by the applied force/stimulus and does not play important role while printing process. Therefore, the control over the micro/nanostructures—stamp interface is key to the successful printing. To this end, control over factors such as surface functionalization, surface morphology modification, temperature and peeling velocity etc. is needed [143–145]. Few recent review papers presented in literature have further described various TP techniques [29, 146, 147]. The TP technology can be exploited to create a pilot line for heterogeneous integration of smart systems in a semiconductor foundry environment for foil-to-foil manufacturing.

Nano/microstructures (NMs, NRs, etc.) of Si and compound semiconductor such as GaAs, GaN, InP, InAs etc. have also been printed using TP approach to produce several classes of flexible devices [45, 46, 48, 87, 96, 108, 136, 141, 148–152]. The TP technique has also been used to transfer carbon-based high mobility materials such as carbon nanotubes (CNTs) and graphene over large area [30, 153–159]. The TP of CNTs is commonly carried out by first depositing a metallic layer (e.g. Au) on top of the CNTs, then, using a transfer substrate typically consisting of PDMS [160], forming a CNTs stamp, and finally, the Au/CNTs layer being transferred to the receiver substrate. TP approach has also been shown for multilayer superstructures of large collections of CNTs configured in horizontally aligned arrays, and complex geometries of arrays and networks on a wide range of substrates [161]. Being a 2-step process, TP has an increased level of complexity when compared to CP. The use of an intermediate stamp introduces some additional challenges. Some of these challenges are discussed in the Sect. 5. Like CP, with cylindrical stamps as shown in Fig. 6e, it may also be possible with TP to have R2R transfer or stamp printing, although this has not been attempted so far [59].

#### Transfer printing of chip or macro scale structures

**Fig. 7:**
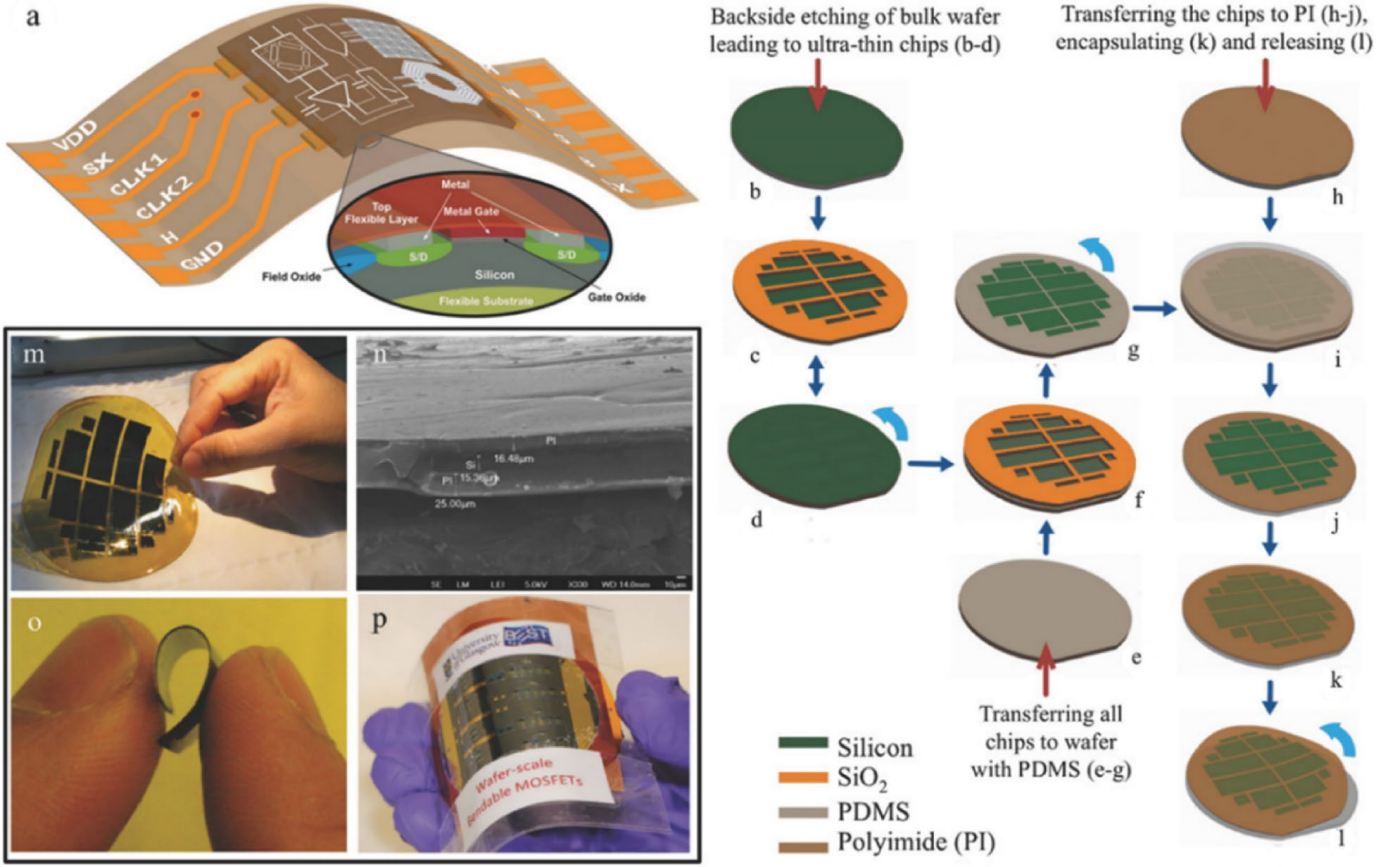
**a**–**p** The process flow of fabrication and transfer of UTCs on to the flexible polyimide substrate through backside chemical etching and PDMS assisted-wafer scale transfer process, respectively. Reprinted with permission from Ref [26]. Copyright (2018) WILEY. **a** Schematic illustration of integrated multilateral stack on flexible substrate. Schematic illustration of backside etching of bulk wafer to UTCs with (**b**) initial bulk wafer, (**c**) back side of UTCs membrane with selective thermal oxide hard mask, and (**d**) the front side. Transfer printing process with two stages: “Stage 1” transferring to carrier substrate with schematic illustration of (**e**) PDMS spin-coated temporary second wafer, (**f**) attaching the UTCs to PDMS, and (**g**) lacer cutting to remove the bulk silicon; “Stage 2” transferring to receiver substrate with schematic illustration of (**h**) polyimide spin-coated on temporary third wafer, (**i**) transfer the UTCs on top of polyimide, (**j**) chemical etching of PDMS and remove the second temporary wafer to expose the top surface, (**k**) spin-coating polyimide to encapsulate the UTCs and (**l**) releasing the temporary third wafer to obtain encapsulated UTCs. **m** Digital image of flexible UTCs transferred on polyimide and (**n**) cross sectional SEM image of UTCs. The photographic image of (**o**) flexible UTCs and (**p**) the n-MOSFET device encapsulated by polyimide

Printed nanomaterials, including NWs, NRs, CNTs and fibers, have been extensively exploited for realizing PE devices but large-scale integration remains an elusive task. UTCs have the capability to fill this gap and deliver high performance flexible electronics with variety of functionalities by combining the high-performance of Si technology with system-in foil applications [26, 49, 103, 128, 162, 163]. At first, the devices and integrated circuits are fabricated on rigid silicon wafer using conventional CMOS approach. Sequentially, thinning as discussed in Sect 2 and transfer printing techniques are utilized to transfer the UTCs to flexible polyimide substrate (Fig. 7) [26, 127, 164, 165]. Figure 7a depicts one of the approaches for TP of UTCs, fabricated via chemical method, on to the flexible polyimide through PDMS assisted wafer scale transfer process [26]. In this transfer process, an oxide layer is thermal grown on rear side of the wafer in selective regions, that acts as hard mask for chemical etching, to achieve UTCs of different dimensions as shown in Fig. 7b–d. Following this, the TP of UTCs on to flexible polyimide is carried out in two stages: (1) transfer to temporary second wafer coated with 200 μm thick PDMS (Fig. 7e–g) and (2) sequential transfer to temporary third wafer coated with polyimide shown in Fig. 7h–l. In stage 1, the 200 μm thick PDMS is spin coated on temporary second silicon carrier wafer and low power plasma treatment is performed to enhance the adhesion of the front side of the silicon membrane to PDMS/temporary wafer. The bulk silicon region is removed by dicing the membrane along the thinned region, which leaves UTCs on PDMS coated second wafer. As the front side of UTCs faces the PDMS (Fig. 7g), stage 2 of transfer process is performed to gain access to the active device by transferring the chip to the receiving substrate (third temporary wafer) coated with polyimide. Sequentially, second temporary wafer is removed by etching the PDMS layer and then UTCs are encapsulated by polyimide on both the sides. Finally, the transfer printed UTCs are released from the third temporary wafer to realize flexible high-performance integrated circuits and devices. The photographic and cross-sectional SEM images of transferred UTCs are shown in Fig. 7m and n, respectively. The flexible UTCs and laminated MOSFET devices between PVC sheets are shown in Fig. 7o and p, respectively [26]. The presented approach opens interesting opportunity for heterogenous integration using organic and inorganic semiconductors on foil. However, thinning of chips makes them fragile, and as a result they require extra care in terms of handling and hence the process for integration can be expensive. Further, the level of flexibility UTCs can achieve is not as high as NWs based PE. For example, the minimum bending radius for UTCs is only ~ 1.4 mm so far [26].

**Fig. 8:**
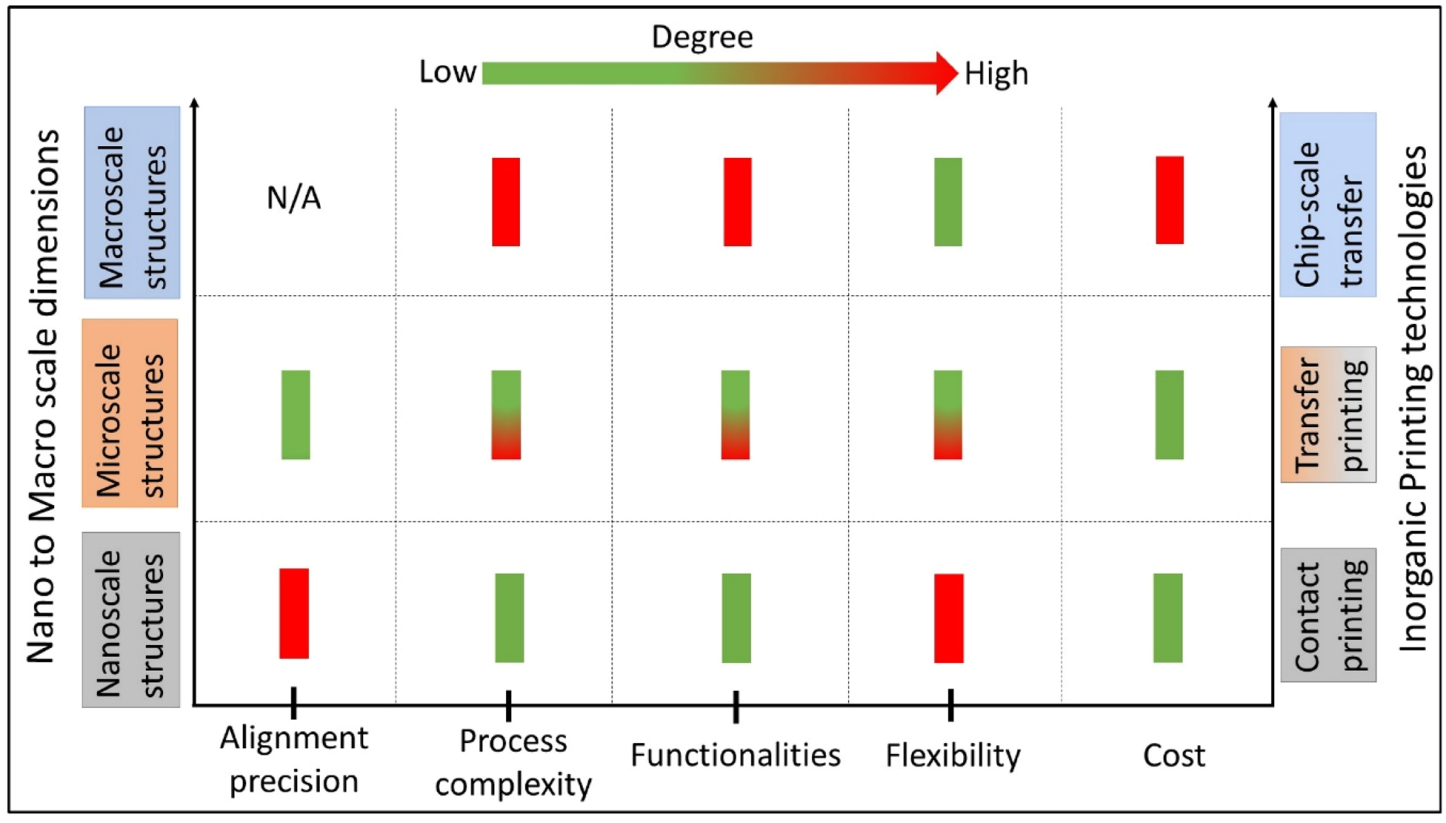
Summary of key features of the printing technologies for the fabrication of flexible large area electronics using diverse inorganic functional inks ranging from nanoscale to macro scale structures. Colour coding is done to show the use of respective printing technology for the transfer of selective scale of inks. For example, contact printing (grey) is generally employed for the transfer of nanoscale structures (grey). N/A, not applicable

Figure 8 summarises key features of the printing technique presented in this section for the fabrication of large area flexible electronics. Exploiting these techniques, nano to chip or macro scale materials has been assembled to obtain devices over large areas, retaining most of the key features of conventional Si-electronics such as short switching time, high integration density, etc. The printing of nanoscale materials, particularly 1D materials with sub-20 nm diameter is of significant interest for printed large area electronics.

## Printed devices/circuits using nano to macro scale elements

This section presents some examples of devices/circuits developed using printed inorganic nano to macro scale structures or building blocks.

### Printed devices

The contact and transfer printing techniques described in previous sections have been utilized to print inorganic structures of various dimensions and materials (described in Sect. 2) to develop devices with flexible and stretchable forms. In general, CP has been used for transfer of nano scale structures (mainly NWs), and TP for nano to macro scale materials. Some of these examples are presented in this section.

#### Transistors

**Fig. 9:**
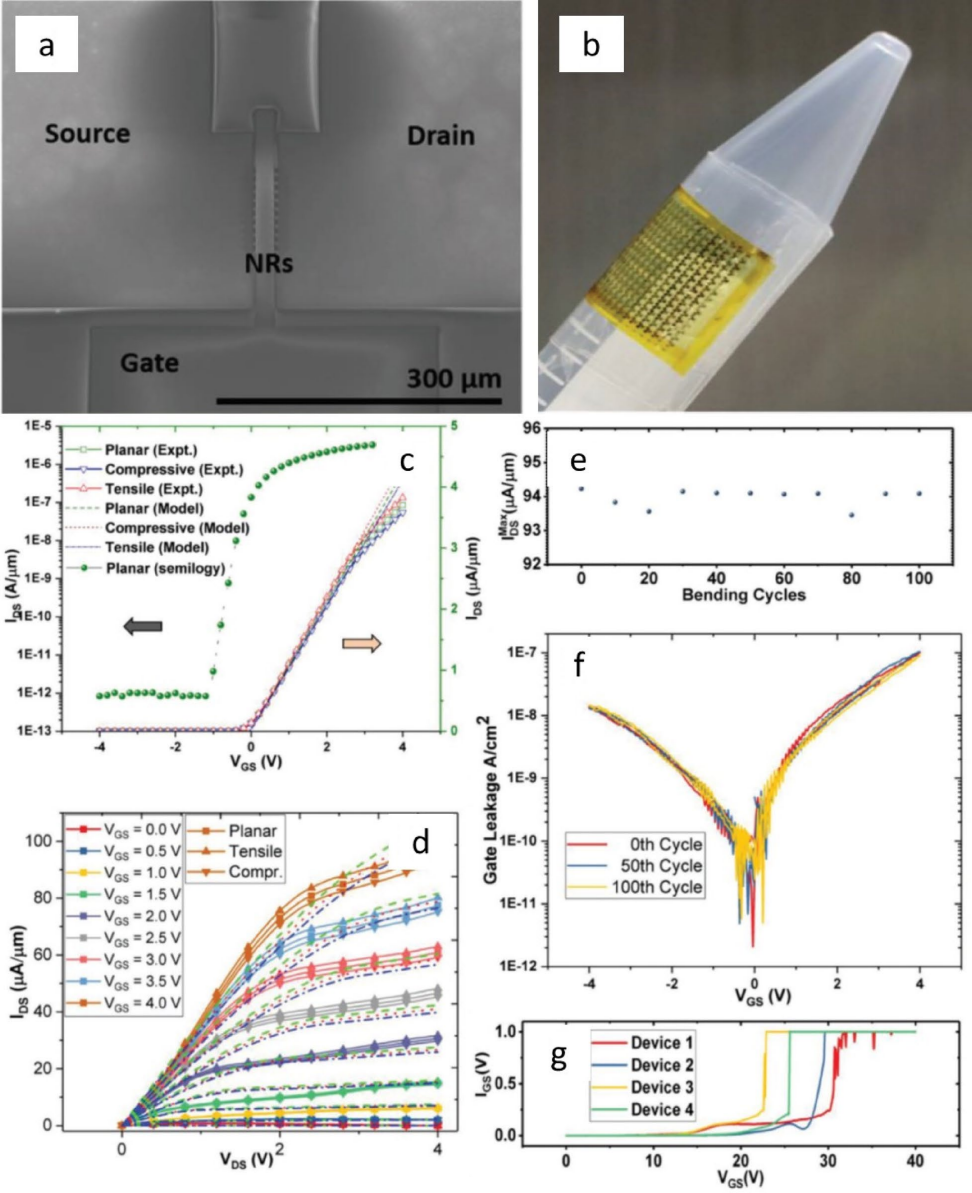
**a** SEM image of a representative NR-FET with the source (S), drain (D), and gate (G) electrodes labelled in it comprising of ten NRs as active layer. **b** Photograph of flexible NR-FETs on PI substrate wrapped on a curved surface. **c** Transfer characteristics (experimental (line) versus model (dashed) simulations) and (**d**) output characteristics of the NR-FET at planar, tensile, and compressive bending conditions (Rc = 40 mm). **e** Variation of the drain current at planar condition after cycles of compressive and tensile bending at V_DS_ = V_GS_ = 4 V. **f** Gate dielectric leakage current Vs gate voltage after subjecting it to cyclic bending. **g** Breakdown voltage characteristics of four randomly chosen devices after subjecting to cyclic bending of 100 cycles. Reproduced with permission from [46]

Generally, effective mobility of an electronic device determines important performance parameters such as switching speed, current density, power efficiency, and transit frequency (*f*_T_) [166]. Compared to the organic thin-film transistors (OTFTs), transistors built on flexible substrates with printed inorganic elements offer a great potential for the emerging application such as IoT where high-performance (e.g. faster communication and computation) is required. NWs have been used to obtain nanoscale electronics devices such as semiconductor NWs assembled into nm-scale FETs [54, 55], and p–n diodes [44, 167]. However, some of the technological processing steps such as deposition of high-quality gate-dielectric, ohmic source/drain contact formation, etc. at room-temperature (RT), are still challenging. Overcoming these challenges, recently, Si-NR-based FETs (NR-FETs) were successfully developed over fully flexible polyimide (PI) substrates, as shown in Fig. 9. The distinct feature of these devices is that the high-quality silicon nitride (SiNx) dielectric was deposited directly on the printed NRs at RT (Fig. 9a–b). The electrical characterisations of NR-FETs have shown high performance (mobility ≈ 656 cm^2^ V^−1^ s^−1^ and on/off ratio > 10^6^) which is on par with the highest performance of similar devices reported with high-temperature processes, and significantly higher than devices reported with low-temperature processes. The reported NR-FETs are mechanically robust, with the ability to withstand mechanical bending cycles (100 cycles tested) without performance degradation (Fig. 9c–f). Generally, ohmicity of the metal–semiconductor (MS) contacts deteriorates with the use of high-temperature dielectric deposition process [168] which affects the transistor performance and its reliability. High performance achieved from NR-FET devices can be attributed to the RT dielectric deposition process with negligible degradation of source/drain contacts. The measured breakdown field strength (> 2.2 MV cm^−1^) further confirms the excellent quality of the RT deposited dielectric (Fig. 9g). High performance transistors based on printed nano/micro scale structures (NWs and NRs) of compound semiconductor such as GaAs, GaN, and InAs on plastic substrates have also been demonstrated, making them potentially useful platforms for ultra-high frequency electronics [45, 98, 169, 170]. Printing of graphene sheets can also produce high-mobility transistors (hole mobility µ_p_ ∼ 3700 cm^2^ V^−1^ s^−1^) [171]. However, one of the limitations of graphene is attributed to its zero-band gap which restricts it from being used in digital applications.

The CP could offer assembly and fabrication of multifunctional 3D NW-based electronics on both planar and flexible substrates through monolithic printing steps [42–44, 172]. In that respect, the 10 functional device layers of Ge/Si NWs, stacked to form a 3D electronic structure are noteworthy [42]. Notably, the NW-FETs show minimal variation in the threshold voltage and exhibit a large average on-current of 4 mA with a standard deviation variation of only 15%. Using CP, multifunctional circuitry that utilizes both the sensory and electronic functionalities of NWs has also been demonstrated, as discussed in the Sect. 4.2.

**Fig. 10:**
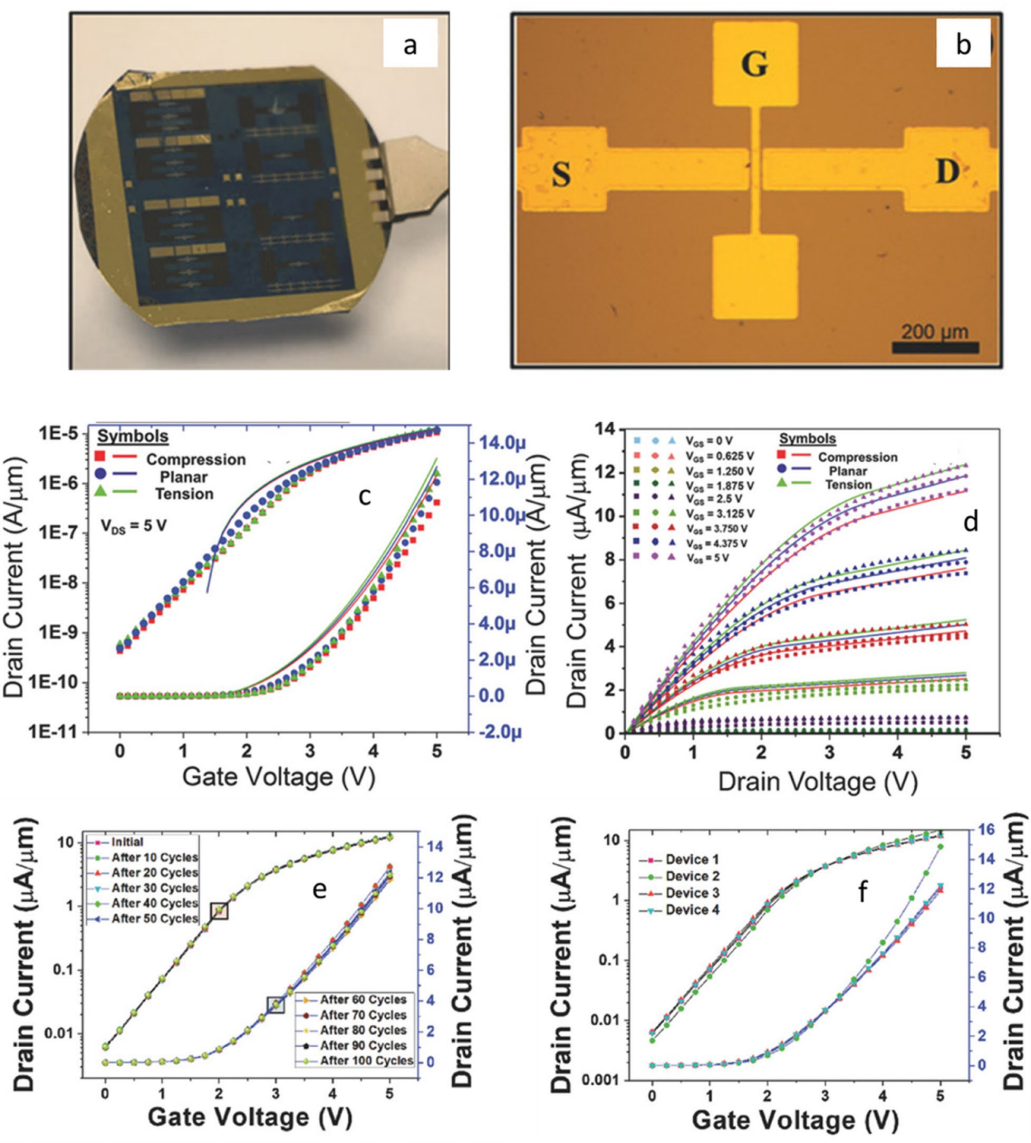
**a** Digital image and (**b**) microscopic image of n-MOSFET. Transistor performance: (**c**) transfer and (**d**) output characteristics of n-MOSFET under various bending strain, (**e**) transfer characteristics under cyclic bending, and (**f**) reliability test with device to device variation. Reprinted with permission from Ref [26]. Copyright (2018) WILEY

Printing of UTCs (chip or macro scale) on flexible substrates is a viable solution to achieve complex or large-scale integrated high-performance electronics with flexible form factor. In this direction, an innovative approach for wafer scale transfer of UTCs on flexible substrates was demonstrated [26]. The methodology is demonstrated with various devices (UTCs with resistive samples [125], metal oxide semiconductor (MOS) capacitors [173–176], n-channel metal-oxide semiconductor field effect transistors (MOSFETs)) [26], CMOS hall sensors [177] and Ion Sensitive Field Effect Transistors (ISFETs) [133]. An example of fabricated MOSFET devices on wafer-scale are shown in Fig. 10a. The microscopic image of a single MOSFET is shown in Fig. 10b. The transfer characteristics of n-MOSFETs are measured under different bending conditions (compressive under concave bending, planar, and tensile under convex bending). Under bending condition, the effective mass of the carrier got affected, due to the splitting and lowering of bands, that results in increase in the overall current under tensile strain and vice versa under compressive strain [133, 162, 163, 178]. This can be observed from the transfer (Fig. 10c) and the output characteristics (Fig. 10d). The n-MOSFET under planar state demonstrated 350 cm^2^/Vs effective mobility and 2.42 × 10^4^ on/off ratio. The effective mobility of the n-MOSFET under tensile and compressive stress are 384 cm^2^/Vs and 333 cm^2^/Vs, respectively. Further, the fabricated device demonstrates stable performance up to 100 bending cycles (Fig. 10e) with negligible device to device variation (Fig. 10f) [26]. This technique has the capability to achieve high-performance flexible circuits with reliable device performance.

#### Photodetectors

**Fig. 11:**
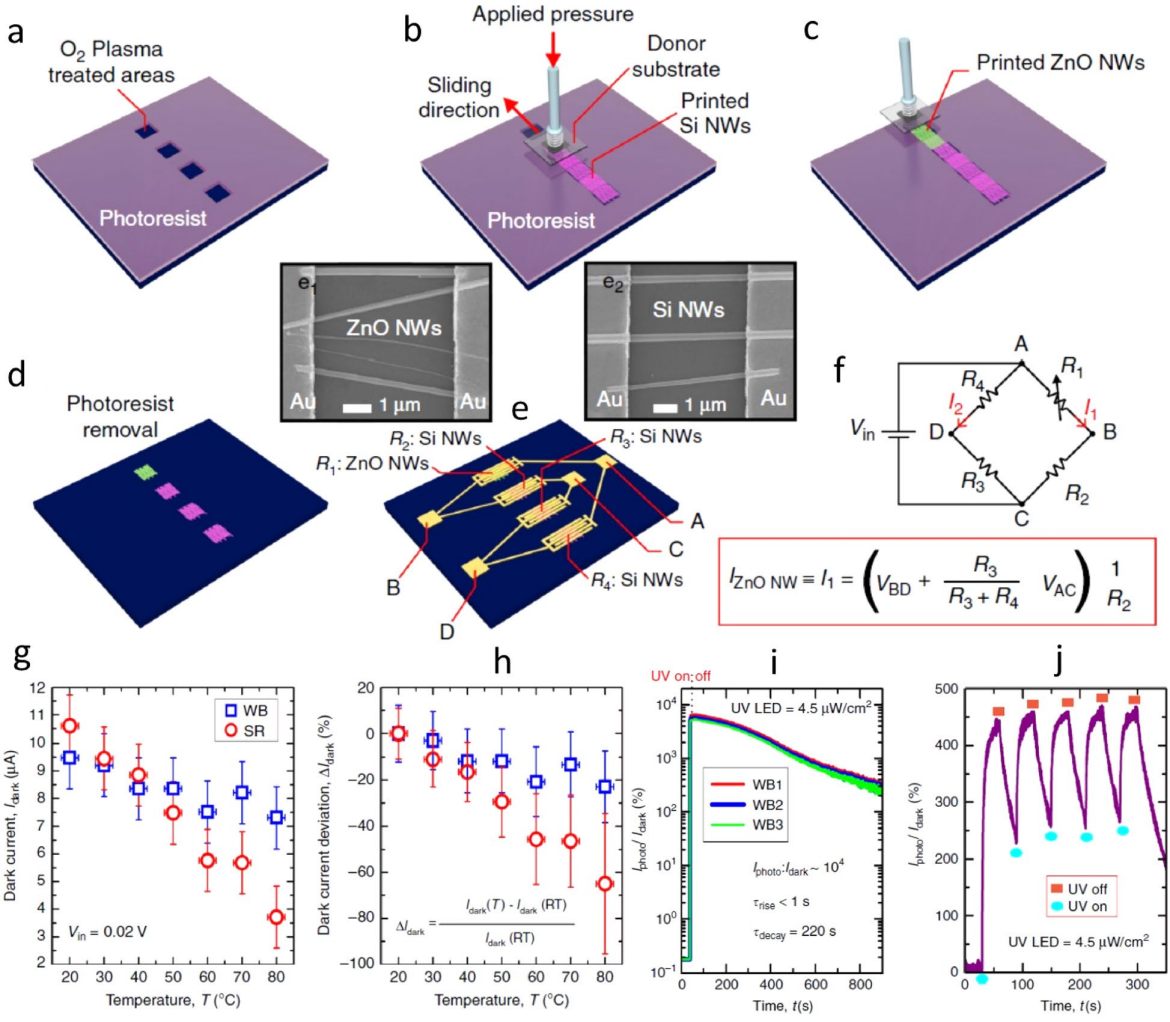
**a**–**e** 3D Schema of UV Photodetector fabrication using contact printed ZnO and Si NWs. Fabrication steps of UV PDs based on ZnO and Si NWs, comprising: (**a**) definition of 20 mm^2^ areas on a S1818 photoresist layer by photolithography, followed by an O2 plasma treatment (100 Watt and 0.3 mbar for 1 min). Contact Printing of (**b**) Si and (**c**) ZnO NWs. **d** removal of the photoresist in warm acetone (50 °C for 2 min). **e** definition of Ti(4 nm)/Au(200 nm) interdigitated electrodes by photolithography and lift-off, where (e1) and (e2) show SEM images of printed ZnO and Si NWs, respectively, bridging a pair of Ti/Au electrodes with a 5 μm gap. **f** WB equivalent circuit and the expression determining the electric current flowing through ZnO NWs. **g** I_dark_ and (**h**) ΔI_dark_ vs. Temp. for WB and single resistance (SR) UV PDs. **i** Single cycle and (**j**) multi-cycles measured over time and using a UV LED power density of 4.5 μW/cm^2^ and a V_in_ of 0.05 V, keeping a distance between UV LED and the PD surface of 5 cm. Reprinted with permission from Ref. [25]

Printing of inorganic building blocks can also be used to form optoelectronic devices such as photodetectors (PDs), light-emitting diodes (LEDs) etc. in a mechanically flexible format [25, 148, 170, 179, 180]. For example, the CP system was used to fabricate ZnO and Si NW-based ultraviolet (UV) PDs with Wheatstone bridge (WB) configuration on rigid and flexible substrates (Fig. 11). The UV PDs based on the printed ensemble of NWs demonstrate high efficiency, a high photocurrent to dark current ratio (> 10^4^) and reduced thermal variations because of inherent self-compensation of WB arrangement. Due to statistically lesser dimensional variations in the ensemble of NWs, the UV PDs made from them have exhibited uniform response. Similarly, printing approaches have been exploited to fabricate visible [43] and near infrared [148] PDs on flexible/stretchable substrates.

#### Energy generators

**Fig. 12:**
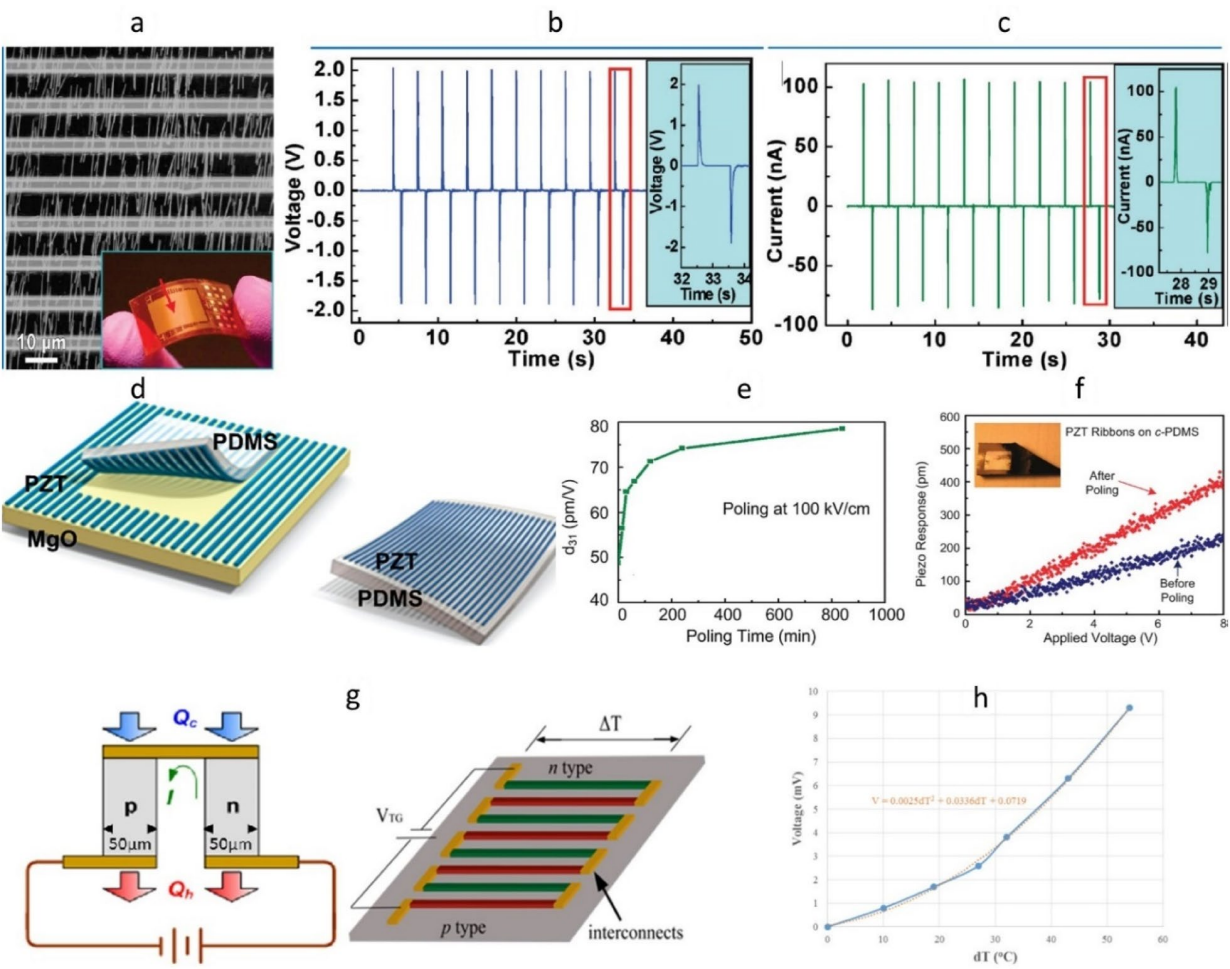
Energy generators fabricated using printed inorganic elements: (**a**–**c**) ZnO NW based piezoelectric nanogenerator (PENG) [184]. **a** SEM image of ZnO NW arrays bonded by Au electrodes. Inset: demonstration of an as-fabricated PENG. The arrowhead indicates the effective working area. **b** Open circuit voltage and (**c**) Short circuit current measurement of the PENG. **d**–**f** NG device made of PZT ribbons formed on a substrate of MgO and then TP onto a sheet of PDMS. **e** d_31_ as a function of poling time at a poling field of ∼ 100 kV/cm. **f** Deflection amplitude vs. modulating AC bias voltage amplitude for PZT ribbons printed on c-PDMS. **g**–**h** Thermoelectric generator (TEG) using Si microwires [82]: **g** scheme of a thermocouple cell, and concept of a TEG module. **h** Graph of open circuit voltage at different temperature gradients

In addition to electronic, and optoelectronic devices, printing of inorganic materials can yield high-performance flexible energy harvesting devices such as solar cells and piezoelectric and thermoelectric generators [82, 124, 182–184]. For example, spatially organized printed ZnO NWs arrays enable fabrication of piezoelectric nanogenerators (PENGs) (Fig. 12a–c). To enhance the output power in PENGs, all NWs must be oriented in same direction. This particular requirement is difficult to be satisfied with most of the nanostructure assembly/integration approaches such as Langmuir–Blodgett (LB) deposition [185], solution shearing methods including blown bubble approach [186, 187], and capillary force assembly [188]. As shown in Fig. 12a, CP method ensure that the crystallographic orientations of the horizontal NWs are aligned along the sweeping direction. Consequently, the polarity of the induced piezopotential is also aligned, leading to a macroscopic potential contributed constructively by all the NWs [184]. Similarly, PZT ribbons were printed using TP approach to fabricate flexible mechanical energy harvester (Fig. 12d–f). Electromechanical characterization of the PZT ribbons by piezo-force microscopy (Fig. 12f) indicates that their energy conversion metrics are among the highest reported on a flexible medium. The TP method was also exploited to develop flexible micro thermoelectric generators (µ-TEGs) on Poly (ethylene terephthalate) (PET) substrate (Fig. 12g–h). A TEG module, consisting of an array of 34 alternately doped p-type and n-type Si microwires, is developed on a SOI wafer using standard photolithography and etching techniques. The TEG modules are transferred from SOI wafer to PET substrate by using TP method. A maximum of 9.3 mV open circuit voltage was recorded from the flexible µ-TEG prototype with a temperature difference of 54 °C.

#### Pressure sensors

**Fig. 13:**
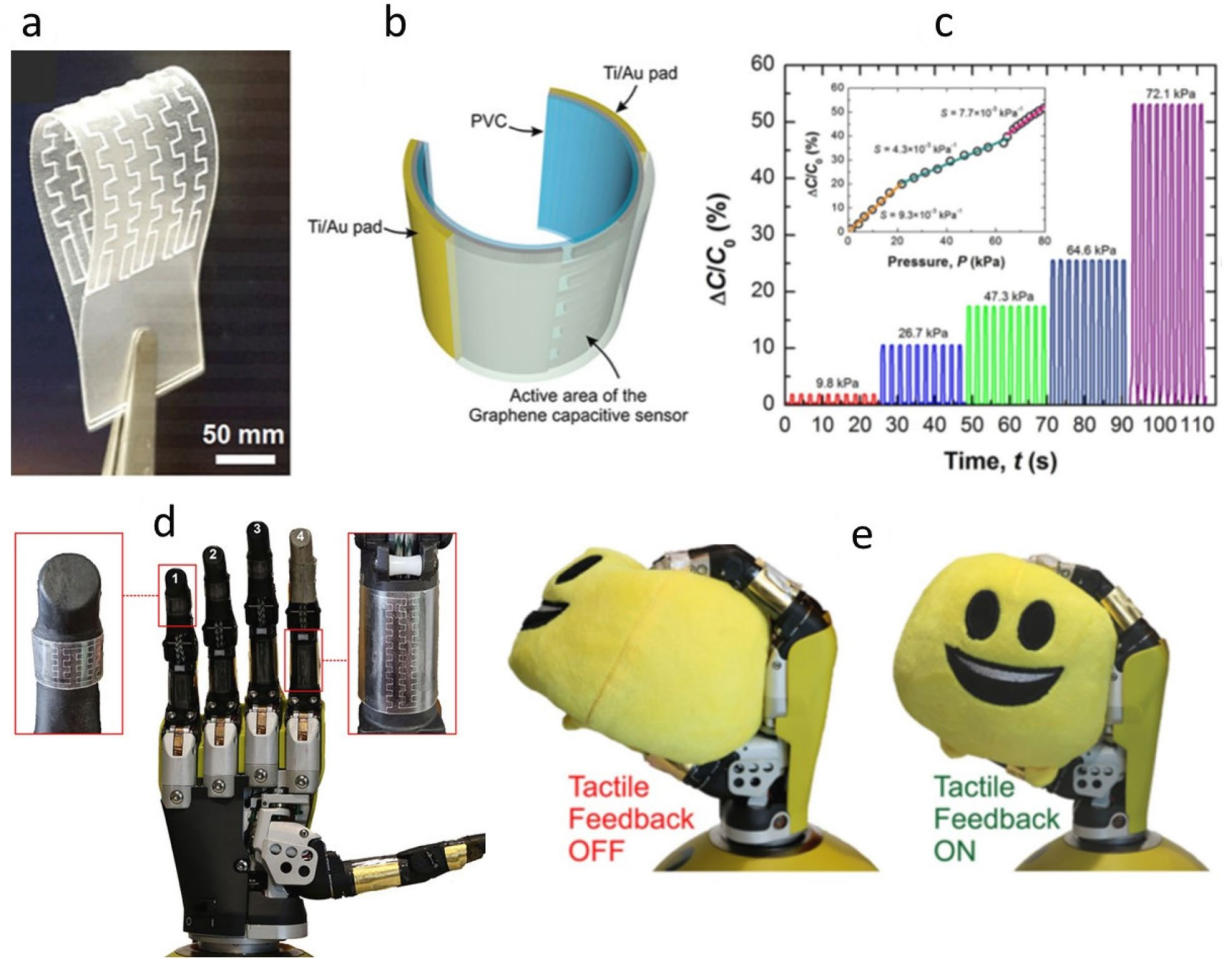
Large area graphene based capacitive tactile sensors: (**a**) Photograph and (**b**) 3D schema of a flexible graphene capacitive touch sensor. **c** Touch sensor response vs. applied pressure. **d** eskin with capacitive sensors integrated onto a robotic hand. **e** Self-powered eskin used as tactile feedback for a robotic hand. Reprinted with permission from Ref. [30]

Large-scale integration of high-performance inorganic nanoscale elements on mechanically flexible substrates enable sensitive sensing devices [30, 189]. For example, large area TP of graphene layer on a photovoltaic (PV) cell resulting in energy autonomous tactile sensitive system for soft robotics (Fig. 13). Transfer of single graphene layer was demonstrated with the transfer of 4-inch CVD grown monolayer of graphene from Cu foil to 125-μm-thick poly vinyl chloride (PVC) substrates by using a hot-lamination method at 125 °C [30]. The transfer process has led to the fabrication of large area flexible graphene based capacitive touch sensors (Fig. 13a–b). The fabricated sensors showed high sensitivity (4.3 kPa^−1^) to a wide range of pressures (0.11–80 kPa) (Fig. 13c). One of the key features of the fabricated eSkin relied on its great transparency, i.e. a sunlight absorption below 5%, which allowed the effective energy harvesting of light energy by a PV cell underneath the eSkin. The viability of graphene-based skin sensors is also analysed by means of a dynamic characterization consisting in the grabbing of a soft object. The response obtained from the capacitive sensors was successfully used as tactile feedback in an artificial hand (Fig. 13d), allowing the manipulation of rigid and soft objects with different shapes (Fig. 13e).

### Printed circuits and systems

**Fig. 14:**
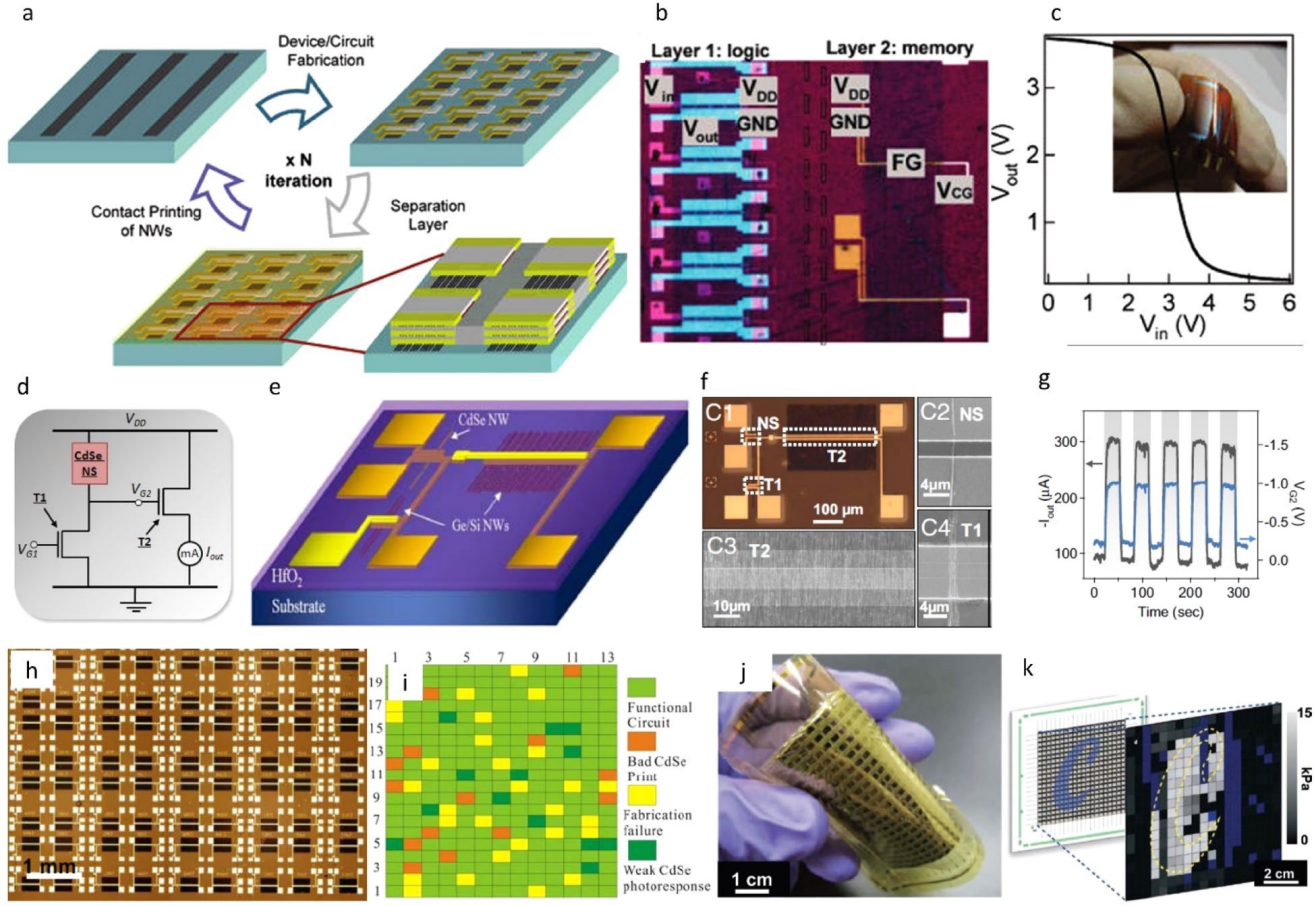
Electronic circuits and systems developed using printed inorganic nano/micro scale structures: (**a**–**c**) 3D NW circuit integration and system [42]. **a** 3D NW circuit is fabricated by the iteration of the contact printing, device fabrication, and separation layer deposition steps N times. **b** Optical image of inverters (layer 1) and floating gate memory (layer 2) on Kapton. **c** DC inverter characteristics. Inset shows functional devices on flexible Kapton substrate. **d**–**i** Heterogeneous NW assembly for an all integrated, sensor circuitry [43]. **d** Circuit diagram for the all-NW PD, with high-mobility Ge/Si NW-FETs (T1 and T2) amplifying the photo response of a CdSe nanosensor. **e** Schematic of the all-NW optical sensor circuit based on ordered arrays of Ge/Si and CdSe NWs. **f** An optical image of the fabricated NW circuitry, consisting of a CdSe nanosensor [(C1), and (C2)] and two Ge/Si core/shell NW-FETs [(C3) and (C4)] with channel widths ≈ 300 µm and 1 µm, respectively. Each device element within the circuit can be independently addressed for dynamics studies and circuit debugging. **g** Circuit output current (blue curve) and voltage divider output voltage (grey curve) response to light illumination (4.4 mW/cm^2^). **h** Optical image of an array of all-NW PD circuitry with each circuit element serving as an independently addressable pixel. **i** A defect analysis map showing the functional and defective NW PD circuit elements. **j** Array of pressure sensors on a flexible substrate, with active matrix addressing using printed arrays of semiconductor nanowires (7 cm × 7 cm with a 19 × 18 pixel array). **k** Measured response of the device under compression in the geometry of a ‘C’ character. The blue pixels represent defects. Reprinted with permission from Ref [189]

Large-scale and heterogeneous integration of printed inorganic nano to macro scale elements have led to the realisation of flexible electronic logic devices, circuits, and systems [39, 42, 43, 141, 189–192]. In one example, using 3D stacking methodology with contact printed NWs (see Sect. 3.1) leads to ultra-high-performance electronics not accessible by scaled complementary metal–oxide–semiconductor (CMOS) (Fig. 14a) [42]. By repeating the printing process, up to ten layers of active NW-FET devices were assembled, and a bilayer structure consisting of logic in layer 1 and non-volatile memory in layer 2 was demonstrated (Fig. 14b–c). In another example, exploiting the sensory and electronic functionalities of nanoscale elements, multifunctional circuitry was realised using contact printed ordered and parallel arrays of optically active CdSe NWs and high-mobility Ge/Si NWs (Fig. 14d–g) [43]. The NW based photo sensors and electronic devices are then interfaced to enable an all-NW circuitry with on-chip integration, capable of detecting and amplifying an optical signal with high sensitivity and precision (Fig. 14h). It was found that ~ 80% of the circuits demonstrated successful photo response operation (Fig. 14i). The potential of CP technique was further demonstrated by large area (7 × 7 cm^2^) printing of parallel NW arrays as the active-matrix backplane of a flexible pressure-sensor array (18 × 19 pixels) (Fig. 14j–k) [189]. The integrated sensor array effectively functions as an eSkin capable of monitoring applied pressure profiles with high spatial resolution. The mechanical flexibility of one such fabricated device can be seen from an optical image shown in Fig. 14j while Fig. 14k shows a pressure map of the same device. The eSkin system can provide fast mapping of normal pressure distributions in the range from 0 and 15 kPa.

## Opportunities and challenges

### Opportunities

As presented in this survey, the last decade has witnessed huge progress in the field of flexible inorganic PE [21, 30, 37, 100, 175, 176, 193–196]. It carries the advantages of both conventional electronics (high performance and functionalities) and printed organic electronics (low-fabrication cost, large area, etc.). Printing of intrinsically flexible high mobility materials such as silicon-based materials (NMs) [48], NRs [46, 103], NWs [25], MWs [100, 123], carbon-based materials (CNTs [197, 198], graphene [30, 176]), two-dimensional (2D) materials of transition-metal dichalcogenides (TMDCs) [199, 200], and metal oxide nanomaterials (such as ZnO) [25, 83, 110, 111, 201, 202] have been attempted. Vertically aligned NWs are usually transferred using CP technique [25], whereas transfer of laterally aligned structures such as NMs and NRs is generally performed using TP approach [46]. Exploiting these printing methods, high mobility materials/inks are used to fabricated variety of flexible electronic devices such as FETs [46, 203], PDs [25, 204], temperature and pressure sensors [205, 206], radio frequency identification tags (RFID) [207], energy harvesters (solar cells, thermoelectric generators, piezoelectric generators, etc.) [82, 148, 182, 184, 208, 209], stretchable interconnects [210] and many others [29, 73]. These intrinsic stretchable inorganic materials have enabled many novel applications that were impossible for conventional electronics as well as for organic PE to achieve such as personal healthcare monitoring [17, 207, 211], human–machine interface (artificial intelligence) [59, 212, 213], neuromorphic computing [140, 214, 215], etc. where faster computing and mechanical flexibility is needed. For the continuous growth of inorganic PE, exploration of the fundamental device physics [30, 140], effects of bending devices [125, 163], innovative fabrication approaches and new form factors required to meet the needs of this next-generation of high-performance large area electronics.

**Fig. 15:**
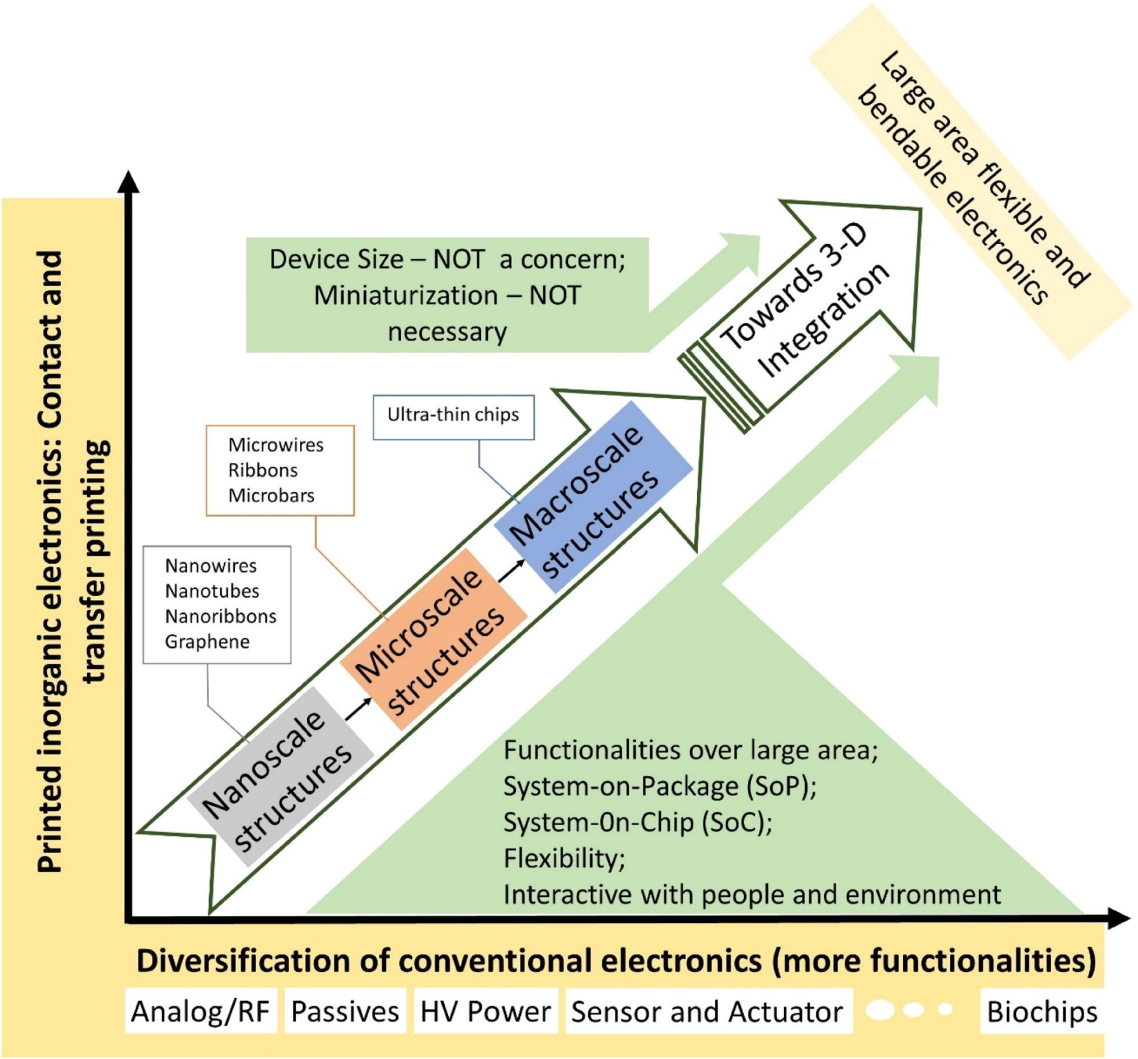
Printed electronics enabled higher diversification and functionalities than conventional electronics including biochips, microelectromechanical sensors, power electronic, analog/RF devices in large area electronics. Printing technologies developed and exploited to provide versatile routes for assembly of nano to macro scale to 3D integration of inorganic functional materials/inks into well-organized arrangements onto various substrates for large area, high performance, flexible inorganic electronics

Large area flexible electronics is mechanically conformable with the human body, enabling human-interactive electronics. Unlike conventional electronics which aims at realizing electronic devices of smaller size and higher density (Moore’s law), the priority of large-area flexible electronics is to fabricate these components with diversified functionalities, such as biochips, microelectromechanical sensors, power electronic, analog/RF devices, flexibility, stretchability, disposability etc. in a cost-effective manner (Fig. 15). Consequently, large area electronics will increasingly be the key for futuristic applications.

Inorganic PE manufacturing has the advantage of being simple and cost-effective approach to provide long-term solutions for large area electronics. The presented survey shows that extensive research effort has been devoted to the development of printing technologies, from research on materials and devices, to fully integrated systems. Printing technologies, mainly contact and transfer printing, facilitates the transfer, assembly and patterning of intrinsically stretchable electronic nanomaterials have been actively investigated and have provided many notable breakthroughs for the advancement of large area electronics (Fig. 8). From the fabrication standpoint, a notable feature of CP and TP methodologies is that they separate semiconductor growth process (rigid substrate) from device (flexible) substrate. The advantage of doing so is the independence of these methods from traditional requirements for epitaxy and thermal budget, which allows the development of transistors, sensors, etc. at temperatures compatible with plastic substrates and that too without sacrificing the ability to incorporate high-quality single crystal semiconductor building blocks. However, several technical challenges exist such as non-uniformity in material growth and its transfer, limited scalability, integration issues including heterogeneous and in three-dimensional which needs to be addressed for next generation of high-performance large area electronics using printing technologies. Some of these challenges are discussed in the following section.

### Challenges

#### Large scale integration of nanoscale features

CP technique has been successful in transferring nanoscale features such as NWs at wafer scale [42, 43], but it is hard to achieve high yield of functional devices. Future printing techniques should be able to transfer nanoscale structures such as NWs and NTs at wafer scale (and larger than wafers) in a controlled manner. To achieve large area printing of these structures, many existing barriers need to be overcome. The foremost is the uniform growth of nanoscale structures over large areas. Top-down growth approach such as using optical and electron beam lithography enabled wet/dry etching consistently demonstrate their superiority in the nanometre control of device definition and placement [77]. On the other hand, bottom-up NW growth approach allow routes to obtain nano features that may not be formed by top-down means. The exact synthesis route to future semiconductor nanostructure-based flexible electronic devices is unclear however, it is quite probable that the route will exploit both top-down and bottom-up techniques in tandem to allow a scalable process to achieve nanostructure at wafer level with good uniformity [88]. The second barrier for large scale integration of nanoscale structures is to develop printing technique that allow transfer of these structures with good uniformity over large area. As a potential solution to non-uniformity issue, CP can be a complementary technique for stamp-printing. This means that CP can be used to print NWs from the growth substrate to a foreign receiver substrate, resulting in a highly aligned arrays of NWs horizontally printed on the receiver substrate. Then, TP can be employed to transfer NWs to the final device substrate. However, the total transfer yield obtained by combining contact- and stamp-printing techniques could be lower than when using CP alone. The main challenge is to achieve a high 100% yield without missing any inks/objects as the destination substrate area increases. To exploit the potential of CP for large area printing, NWs can be directly grown on cylindrical rolls using bottom-up process which can be used as a stamp (Fig. 6c) [59]. The bottom-up synthesis of NWs on tubes of glass, quartz, and stainless-steel and even on polymers like PDMS, has been demonstrated in the past [53, 83]. One could see new commercial opportunities, for example, commercializing NW rolls just as the Si wafers today. By using such rolls in differential roll printing [53] and roll transfer-printing [172] settings, the CP approach can be extended to an R2R-type printing.

#### Technological parameters

The technological parameters such as channel lengths, ohmic junctions etc. are also important factors influencing the device performance. TP of micro/nano structures, produced from the parent wafer using standard microfabrication techniques, results in well-defined structures over target device substrates. However, residues from the intermediate stamp may remain on the surface of the micro/nanostructures which are transferred on the target substrate [99, 130]. The interfacial contact between the active micro/nanostructures and deposited metal contacts or dielectric material need attention as they play critical role in the electrical performance and reliability of the device. Since the elastomeric stamp is generally an insulating material (e.g. PDMS), its residue may pose a challenge in employing the transferred nanostructures as building block for high performance electronics. For example, in presence of PDMS residues it is difficult to realize metal contacts for the source and drain terminals of a Si micro/nano wire transistor. The reported method [99] provides the solution of achieving a complete removal of PDMS residues from the surface of transferred micro/nanostructure on flexible substrate. Moreover, considering the micro/nanostructure dimensions such as NWs, NRs, etc., transfer steps are more complex. A perfect mask alignment for such diminished size may pose challenges. Nevertheless, using TP approach, the multistep stamp printing has been successfully demonstrated with feature resolution down to nanoscale [216]. Another challenge is the printing of high-resolution, high-aspect-ratio metal lines for the miniaturisation of device channel length. The channel length is a very critical parameter in CMOS technology for high device performance. At present, printed transistors have channel length in few microns which is far larger than the advanced conventional Si electronics (few nanometres). However, during the initial development stages i.e. the time when CMOS technologies were at the point where PE presently is, the channel length was longer than 1 µm. Going with the growth trend for CMOS technologies, directly printing submicron channel on printed inorganic semiconductors could be possible in future with advances in printed technologies.

#### Direct 3D integration capability

The 3D integration of PE could offer major advantages in the future for miniaturized high-performance flexible devices, just like the 3D integration of conventional CMOS electronics. The CP technique has shown potential to be used for vertical 3D stacking of printed NW [42]. As an example, functional device in 10 vertically stacked of Ge/Si NWs have been shown [42]. The best attribute of CP is the compatibility with monolithic 3D integration, which means that layer-by-layer assembly does not alter the properties of existing layers. Moreover, as mentioned previously, CP and TP can complement each other in layer-by-layer printing of NWs forming vertical 3D stacking. The advances in multi-material additive manufacturing could offer new avenues for introducing eSkin like features in prosthesis and robotics [59, 217, 218]. For instance, such 3D manufacturing processes could be employed to develop prosthesis with directly integrated or embedded touch sensors, thereby enabling robust limbs that are also free from wear and tear issues. The ability to simultaneously print multiple materials in 3D will also address the traditional robotic eSkin issue of routing of wiring.

#### Heterogeneous integration of materials for multi-functionality

Bringing multi-functionality into eSkin like devices or other wearables is important for efficient miniaturization and monitoring of different input parameters using single device [14, 43, 170, 219]. The heterogeneously integrated NWs with distinct functionality will represent the future technology, where cost-competitive, scalable strategies allow integration of diverse materials with complementary performance [25, 43, 220–222]. The need of the hour is to develop printing techniques overcoming the critical issue of multi-functionality, and permitting the highly precise integration of individually selected semiconductor NWs from different materials (e.g. InP, GaAs, ZnO, Si) onto a variety of substrates (e.g. polymer, silicon, silica, metals) [25]. This will open avenues towards the manufacture of heterogeneous devices, consisting of integrated systems made from pure and/or hybrid inorganic/organic materials.

## Conclusions

PE technologies are emerging as a dynamic manufacturing route for large area high-performance electronics. This advancement is compelled by the demand for new functionalities such as flexible, conformal devices for application in wearables, robotics, healthcare etc. However, modest performances thus far offered by organic semiconducting and dielectric materials-based inks has restricted PE applications towards low-end. To this end, printed inorganic semiconducting materials-based devices show huge potential to achieve performance at par with silicon-based electronics. The presented survey captures the recent developments in the field of inorganic PE. Key printing techniques to integrate nano to macro scale inorganic functional elements are presented. Each transfer technique has distinct advantages and disadvantages depending on many factors: ink structure, orientation, and dimensions, and application requirements (flexibility, functionality and so on). The advancements in PE technologies has essentially enlarged the range of high-performing materials that can be patterned onto variety of non-conventional substrates to achieve new form factors including stretchability. At last, we have discussed challenges and potential solution for nanostructure-based large area high-performance electronics, mainly due to their simplicity, low processing temperatures, suitability for large-area and mass production (compatibility with R2R technology), compatibility with 2D and 3D monolithic integration, reproducibility, reliability and compatibility with flexible substrates. Advancement in inorganic printed electronics open avenues for complex circuits/devices fabrication with CMOS comparable performances, enabling new circuit topologies, heterogeneous integration, and will increasingly interact with their environment. The unification of new form factors, diversification and functionality is an appealing new aspect for electronics manufacturing and can be achieved by printing techniques.

## Data Availability

Data sharing is not applicable to this article as no datasets were generated or analysed during the current study.

## Abbreviations

PE: Printed Electronics
CP: Contact Printing
TP: Transfer Printing
UTCs: Ultra-thin chips
R2R: Roll-to-roll
NMs: Nanomembranes
NRs: Nanoribbons
NWs: Nanowires
CNTs: Carbon nanotubes
NT: Nanotube
2D: Two-dimensional
3D: Three-dimensional
TMDCs: Transition-metal dichalcogenides
FETs: Field-effect transistors
RFID: Radio frequency identification tags
VLS: Vapour–liquid–solid
VS: Vapour–solid
VSS: Vapour–solid–solid
MACE: Metal-assisted chemical etching
CMOS: Complementary metal–oxide–semiconductor
PDMS: Polydimethylsiloxane
SOI: Silicon-on-insulator
BOX: Buried oxide
IPA: Iso-propyl alcohol
RIE: Reactive ion etching
DI: Deionised
PECVD: Plasma enhanced chemical vapour deposition
SOD: Spin-on-dopant
HEMTs: High electron mobility transistors
MESFETs: Metal-semiconductor field effect transistors
MOSFETs: Metal-oxide semiconductor field-effect transistors
PI: Polyimide
TFTs: Thin-film transistors
NR-FETs: Nanoribbon field-effect transistors
NM-FET: Nanomembrane field-effect transistors
PVC: Poly vinyl chloride
CST: Controlled spalling technique
TMAH: Tetramethyl ammonium hydroxide

## References

[1] Song E, Li J, Won SM, Bai W, Rogers JA (2020). Nat. Mater..

[2] Lee K, Ni X, Lee JY, Arafa H, Pe DJ, Xu S, Avila R, Irie M, Lee JH, Easterlin RL, Kim DH, Chung HU, Olabisi OO, Getaneh S, Chung E, Hill M, Bell J, Jang H, Liu C, Park JB, Kim J, Kim SB, Mehta S, Pharr M, Tzavelis A, Reeder JT, Huang I, Deng Y, Xie Z, Davies CR, Huang Y, Rogers JA (2020). Nat. Biomed. Eng..

[3] Hassani FA, Jin H, Yokota T, Someya T, Thakor NV (2020). Sci. Adv..

[4] Chow PCY, Someya T (2020). Adv. Mater..

[5] Jiang C, Choi HW, Cheng X, Ma H, Hasko D, Nathan A (2019). Science..

[6] Lin Z, Huang Y, Duan X (2019). Nat. Electron..

[7] Hosseini ES, Manjakkal L, Shakthivel D, Dahiya R, Appl ACS (2020). Mater. Interfaces.

[8] Kafi MA, Paul A, Vilouras A, Hosseini ES, Dahiya RS (2020). IEE Sens. J..

[9] Yang JC, Mun J, Kwon SY, Park S, Bao Z, Park S (2019). Adv. Mater..

[10] Niu S, Matsuhisa N, Beker L, Li J, Wang S, Wang J, Jiang Y, Yan X, Yun Y, Burnett W, Poon ASY, Tok JB-H, Chen X, Bao Z (2019). Nat. Electron..

[11] H. U. Chung, B. H. Kim, J. Y. Lee, J. Lee, Z. Xie, E. M. Ibler, K. Lee, A. Banks, J. Y. Jeong, J. Kim, C. Ogle, D. Grande, Y. Yu, H. Jang, P. Assem, D. Ryu, J. W. Kwak, M. Namkoong, J. Bin Park, Y. Lee, D. H. Kim, A. Ryu, J. Jeong, K. You, B. Ji, Z. Liu, Q. Huo, X. Feng, Y. Deng, Y. Xu, K.-I. Jang, J. Kim, Y. Zhang, R. Ghaffari, C. M. Rand, M. Schau, A. Hamvas, D. E. Weese-Mayer, Y. Huang, S. M. Lee, C. H. Lee, N. R. Shanbhag, A. S. Paller, S. Xu, and J. A. Rogers, Science. **363**, (2019)

[12] Ray TR, Choi J, Bandodkar AJ, Krishnan S, Gutruf P, Tian L, Ghaffari R, Rogers JA (2019). Chem. Rev..

[13] Ling Y, An T, Yap LW, Zhu B, Gong S, Cheng W (2019). Adv. Mater..

[14] Bhattacharjee M, Middya S, Escobedo P, Chaudhuri J, Bandyopadhyay D, Dahiya R (2020). Biosens. Bioelectron..

[15] Navaraj W, Dahiya R (2019). Adv. Intell. Syst..

[16] Ozioko O, Karipoth P, Hersh M, Dahiya R (2020). IEEE Trans. Neural Syst. Rehabil. Eng..

[17] Manjakkal L, Dang W, Yogeswaran N, Dahiya R (2019). Biosensors.

[18] Manjakkal L, Navaraj WT, Núñez CG, Dahiya R (2019). Adv. Sci..

[19] Jiang Z, Nayeem MOG, Fukuda K, Ding S, Jin H, Yokota T, Inoue D, Hashizume D, Someya T (2019). Adv. Mater..

[20] Someya T, Amagai M (2019). Nat. Biotechnol..

[21] R. Dahiya, W. T. Navaraj, S. Khan, and E. O. Polat, Inf. Disp. (1975). **31**, (2015)

[22] Khan S, Lorenzelli L, Dahiya RS (2015). IEEE Sens. J..

[23] Gao W, Ota H, Kiriya D, Takei K, Javey A (2019). Acc. Chem. Res..

[24] Yu Y, Nyein HYY, Gao W, Javey A (2020). Adv. Mater..

[25] Núñez CG, Liu F, Navaraj WT, Christou A, Shakthivel D, Dahiya R (2018). Microsystems Nanoeng..

[26] Navaraj WT, Gupta S, Lorenzelli L, Dahiya R (2018). Adv. Electron. Mater..

[27] S. M. F. Cruz, L. A. Rocha, and J. C. Viana, *Printing Technologies on Flexible Substrates for Printed Electronics* (2018)

[28] Wallin TJ, Pikul J, Shepherd RF (2018). Nat. Rev. Mater..

[29] Linghu C, Zhang S, Wang C, Song J (2018). Npj Flex. Electron..

[30] Núñez CG, Navaraj WT, Polat EO, Dahiya R (2017). Adv. Funct. Mater..

[31] Dahiya R (2019). Proc. IEEE.

[32] Koutsiaki C, Kaimakamis T, Zachariadis A, Logothetidis S (2019). Mater. Today Proc..

[33] Vaklev NL, Steinke JHG, Campbell AJ (2019). Adv. Mater. Interfaces.

[34] Fattori M, Fijn J, Harpe P, Charbonneau M, Lombard S, Romanjek K, Locatelli D, Tournon L, Laugier C, Cantatore E (2019). IEEE Electron Device Lett..

[35] Fukuda K, Someya T (2017). Adv. Mater..

[36] Manjakkal L, Pullanchiyodan A, Yogeswaran N, Hosseini E, Dahiya R (2020). Adv. Mater..

[37] Sekitani T, Someya T (2010). Adv. Mater..

[38] Ozer E, Kufel J, Myers J, Biggs J, Brown G, Rana A, Sou A, Ramsdale C, White S (2020). Nat. Electron..

[39] Liu F, Dahiya AS, Dahiya R (2020). Nat. Electron..

[40] Liu A, Zhu H, Noh YY (2019). Mater. Sci. Eng. R Reports.

[41] Manjakkal L, Szwagierczak D, Dahiya R (2020). Prog. Mater Sci..

[42] Javey A, Nam S, Friedman RS, Yan H, Lieber CM (2007). Nano Lett..

[43] Fan Z, Ho JC, Jacobson ZA, Razavi H, Javey A (2008). Proc. Natl. Acad. Sci. U. S. A..

[44] Fan Z, Ho JC, Jacobson ZA, Yerushalmi R, Alley RL, Razavi H, Javey A (2008). Nano Lett..

[45] Sun Y, Kim H-S, Menard E, Kim S, Adesida I, Rogers JA (2006). Small.

[46] A. Zumeit, W. T. Navaraj, D. Shakthivel, and R. Dahiya, Adv. Electron. Mater. 1901023 (2020)

[47] Y. Kumaresan, H. Kim, Y. Pak, P. K. Poola, R. Lee, N. Lim, H. C. Ko, G. Y. Jung, and R. Dahiya, Adv. Electron. Mater. 2000058 (2020)

[48] Han X, Seo KJ, Qiang Y, Li Z, Vinnikova S, Zhong Y, Zhao X, Hao P, Wang S, Fang H (2019). Npj Flex. Electron..

[49] Gupta S, Navaraj WT, Lorenzelli L, Dahiya R (2018). Npj Flex. Electron..

[50] Hurtado A, Jevtics D, Guilhabert B, Gao Q, Tan HH, Jagadish C, Dawson MD (2018). IET Optoelectron..

[51] Guilhabert B, Hurtado A, Jevtics D, Gao Q, Tan HH, Jagadish C, Dawson MD (2016). ACS Nano.

[52] McAlpine MC, Ahmad H, Wang D, Heath JR (2007). Nat. Mater..

[53] Yerushalmi R, Jacobson ZA, Ho JC, Fan Z, Javey A (2007). Appl. Phys. Lett..

[54] Zheng G, Lu W, Jin S, Lieber CM (2004). Adv. Mater..

[55] Xiang J, Lu W, Hu Y, Wu Y, Yan H, Lieber CM (2006). Nature.

[56] Oshima Y, Nakamura A, Matsunaga K (2018). Science..

[57] Li H, Cao Y, Wang Z, Feng X (2019). Opt. Mater. Express.

[58] Yu KJ, Yan Z, Han M, Rogers JA (2017). Npj Flex. Electron..

[59] Dahiya R, Yogeswaran N, Liu F, Manjakkal L, Burdet E, Hayward V, Jorntell H (2019). Proc. IEEE.

[60] Dahiya R, Akinwande D, Chang JS (2019). Proc. IEEE.

[61] Wu W (2017). Nanoscale.

[62] Zschieschang U, Klauk H (2019). J. Mater. Chem. C.

[63] Matsui H, Takeda Y, Tokito S (2019). Org. Electron..

[64] Ling H, Liu S, Zheng Z, Yan F (2018). Small Methods.

[65] Chang JS, Facchetti AF, Reuss R (2017). IEEE J. Emerg. Sel. Top. Circuits Syst..

[66] Berggren M, Nilsson D, Robinson ND (2007). Nat. Mater..

[67] Lou Z, Shen GZ (2015). Adv. Sci..

[68] Liu Z, Xu J, Chen D, Shen G (2015). Chem. Soc. Rev..

[69] S. S. Su and I. Chang, in *Commer Nanotechnologies*–*A Case Study Approach*, ed. by D. Brabazon, E. Pellicer, F. Zivic, J. Sort, M. D. Baró, N. Grujovic, and K.-L. Choy (Springer International Publishing, Cham, 2018), pp. 15–29

[70] Shakthivel D, Ahmad M, Alenezi MR, Dahiya R, Silva SRP (2019). Cambridge.

[71] S. Chung, K. Cho, and T. Lee, Adv. Sci. **6**, (2019) 10.1002/advs.201801445 PMC6425446 30937255

[72] Huang Q, Zhu Y (2019). Adv. Mater. Technol..

[73] Carlson A, Bowen AM, Huang Y, Nuzzo RG, Rogers JA (2012). Adv. Mater..

[74] Ejaz A, Han JH, Dahiya R (2020). J. Colloid Interface Sci..

[75] Shakthivel D, Navaraj WT, Champet S, Gregory DH, Dahiya RS (2019). Nanoscale Adv..

[76] C. G. Núñez, F. Liu, S. Xu, and R. Dahiya, Cambridge, U.K. Cambridge Univ. Press **4015**, (2018)

[77] Hobbs RG, Petkov N, Holmes JD (2012). Chem. Mater..

[78] Yu H-D, Regulacio MD, Ye E, Han M-Y (2013). Chem. Soc. Rev..

[79] Opoku C, Dahiya AS, Oshman C, Daumont C, Cayrel F, Poulin-Vittrant G, Alquier D, Camara N (2015). Nanotechnology.

[80] Boubenia S, Dahiya AS, Morini F, Nadaud K, Alquier D (2017). Sci. Rep..

[81] Sun H, Li X (2019). Phys. Status Solidi.

[82] S. Khan, R. S. Dahiya, and L. Lorenzelli, Eur. Solid-State Device Res. Conf. 86 (2014)

[83] Dahiya AS, Morini F, Boubenia S, Nadaud K, Alquier D, Poulin-Vittrant G (2018). Adv. Mater. Technol..

[84] Nadaud K, Morini F, Dahiya AS, Justeau C, Boubenia S, Rajeev KP, Alquier D, Poulin-Vittrant G (2018). Appl. Phys. Lett..

[85] Graton O, Poulin-Vittrant G, Dahiya AS, Camara N, Hue L-PTH, Lethiecq M (2013). Phys. Status Solidi Rapid Res. Lett..

[86] Sun Y, Graff RA, Strano MS, Rogers JA (2005). Small.

[87] Sun Y, Rogers JA (2004). Nano Lett..

[88] Núñez CG, Navaraj WT, Liu F, Shakthivel D, Dahiya R, Appl ACS (2018). Mater. Interfaces.

[89] Dejarld M, Shin JC, Chern W, Chanda D, Balasundaram K, Rogers JA, Li X (2011). Nano Lett..

[90] Choi WK, Liew TH, Dawood MK, Smith HI, Thompson CV, Hong MH (2008). Nano Lett..

[91] De Boor J, Geyer N, Wittemann JV, Gösele U, Schmidt V (2010). Nanotechnology.

[92] Damilano B, Coulon P-M, Vézian S, Brändli V, Duboz J-Y, Massies J, Shields PA (2019). Appl. Phys. Express.

[93] K. Kim, J. K. Lee, S. J. Han, and S. Lee, Appl. Sci. **10**, (2020)

[94] Naureen S, Sanatinia R, Shahid N, Anand S (2011). Nano Lett..

[95] Fernández-Garrido S, Auzelle T, Lähnemann J, Wimmer K, Tahraoui A, Brandt O (2019). Nanoscale Adv..

[96] Sun Y, Kim S, Adesida I, Rogers JA (2005). Appl. Phys. Lett..

[97] Menard E, Nuzzo RG, Rogers JA (2005). Appl. Phys. Lett..

[98] Ahn JH, Kim HS, Lee KJ, Zhu Z, Menard E, Nuzzo RG, Rogers JA (2006). IEEE Electron Device Lett..

[99] Dahiya R, Gottardi G, Laidani N (2015). Microelectron. Eng..

[100] Khan S, Lorenzelli L, Dahiya R (2016). IEEE J. Electron Devices Soc..

[101] Mack S, Meitl MA, Baca AJ, Zhu Z-T, Rogers JA (2006). Appl. Phys. Lett..

[102] Ko HC, Baca AJ, Rogers JA (2006). Nano Lett..

[103] Baca AJ, Ahn J-H, Sun Y, Meitl MA, Menard E, Kim H-S, Choi WM, Kim D-H, Huang Y, Rogers JA (2008). Angew. Chemie Int. Ed..

[104] Yang Z, Surrente A, Tutuncuoglu G, Galkowski K, Cazaban-Carrazé M, Amaduzzi F, Leroux P, Maude DK, Morral AFI, Plochocka P (2017). Nano Lett..

[105] Bollani M, Fedorov A, Albani M, Bietti S, Bergamaschini R, Montalenti F, Ballabio A, Miglio L, Sanguinetti S (2020). Crystals.

[106] Raya AM, Friedl M, Martí-Sánchez S, Dubrovskii VG, Francaviglia L, Alén B, Morgan N, Tütüncüoglu G, Ramasse QM, Fuster D, Llorens JM, Arbiol J, Morral AFI (2020). Nanoscale.

[107] Liu J, Usami K, Naesby A, Bagci T, Polzik ES, Lodahl P, Stobbe S (2011). Appl. Phys. Lett..

[108] Lee KJ, Lee J, Hwang H, Reitmeier ZJ, Davis RF, Rogers JA, Nuzzo RG (2005). Small.

[109] Yang W, Yang H, Qin G, Ma Z, Berggren J, Hammar M, Soref R, Zhou W (2010). Appl. Phys. Lett..

[110] Dahiya AS, Opoku C, Poulin-Vittrant G, Camara N, Daumont C, Barbagiovanni EG, Franzò G, Mirabella S, Alquier D, Appl ACS (2017). Mater. Interfaces.

[111] Dahiya AS, Opoku C, Alquier D, Poulin-Vittrant G, Cayrel F, Graton O, Hue L-PTH, Camara N (2014). Nanoscale Res. Lett..

[112] Hannon JB, Kodambaka S, Ross FM, Tromp RM (2006). Nature.

[113] Núñez CG, Pau JL, Ruíz E, Marín AG, García BJ, Piqueras J, Shen G, Wilbert DS, Kim SM, Kung P (2014). Thin Solid Films.

[114] Fan HJ, Bertram F, Dadgar A, Christen J, Krost A, Zacharias M (2004). Nanotechnology.

[115] Dai ZR, Pan ZW, Wang ZL (2003). Adv. Funct. Mater..

[116] Scott CD, Arepalli S, Nikolaev P, Smalley RE (2001). Appl. Phys. A.

[117] Persson AI, Larsson MW, Stenström S, Ohlsson BJ, Samuelson L, Wallenberg LR (2004). Nat. Mater..

[118] Lensch-Falk JL, Hemesath ER, Perea DE, Lauhon LJ (2009). J. Mater. Chem..

[119] Thombare SV, Marshall AF, McIntyre PC (2013). APL Mater..

[120] Cui H, Lü YY, Yang GW, Chen YM, Wang CX (2015). Nano Lett..

[121] Sun Q, Pan D, Li M, Zhao J, Chen P, Lu W, Zou J (2020). Nanoscale.

[122] Polat EO, Balci O, Kakenov N, Uzlu HB, Kocabas C, Dahiya R (2015). Sci. Rep..

[123] Khan S, Yogeswaran N, Taube W, Lorenzelli L, Dahiya R (2015). J. Micromechanics Microengineering.

[124] Qi Y, McAlpine MC (2010). Energy Environ. Sci..

[125] Dahiya RS, Gennaro S (2013). IEEE Sens. J..

[126] Hussain AM, Hussain MM (2016). Adv. Mater..

[127] Shahrjerdi D, Bedell SW (2013). Nano Lett..

[128] Dong Z, Lin Y (2020). Mater. Sci. Semicond. Process..

[129] Lin Y, Yuan R, Zhang X, Chen Z, Zhang H, Su Z, Guo S, Wang X, Wang C (2019). Silicon.

[130] Gupta S, Vilouras A, Dahiya R (2020). Microelectron. Eng..

[131] Tea NH, Milanovic V, Zincke CA, Suehle JS, Gaitan M, Zaghloul ME, Geist J (1997). J. Microelectromech. Syst..

[132] Kim YS, Maeda N, Kitada H, Fujimoto K, Kodama S, Kawai A, Arai K, Suzuki K, Nakamura T, Ohba T (2013). Microelectron. Eng..

[133] Vilouras A, Christou A, Manjakkal L, Dahiya R (2020). ACS Appl.

[134] Takahashi T, Takei K, Ho JC, Chueh Y-L, Fan Z, Javey A (2009). J. Am. Chem. Soc..

[135] Lieber CM, Wang ZL (2007). MRS Bull..

[136] Sun Y, Rogers JA (2007). Adv. Mater..

[137] Fan Z, Ho JC, Takahashi T, Yerushalmi R, Takei K, Ford AC, Chueh Y-L, Javey A (2009). Adv. Mater..

[138] Yao J, Yan H, Lieber CM (2013). Nat. Nanotechnol..

[139] Song H, Lee MH (2013). Nanotechnology.

[140] Navaraj WT, Núñez CG, Shakthivel D, Vinciguerra V, Labeau F, Gregory DH, Dahiya R (2017). Front. Neurosci..

[141] Park S, Xiong Y, Kim R, Elvikis P, Meitl M, Kim D, Wu J, Yoon J, Yu C, Liu Z, Huang Y, Hwang K, Ferreira P, Li X, Choquette K, Rogers JA (2009). Science..

[142] Kim-Lee H-J, Carlson A, Grierson DS, Rogers JA, Turner KT (2014). J. Appl. Phys..

[143] Loo Y-L, Willett RL, Baldwin KW, Rogers JA (2002). J. Am. Chem. Soc..

[144] Carlson A, Wang S, Elvikis P, Ferreira PM, Huang Y, Rogers JA (2012). Adv. Funct. Mater..

[145] Yang SY, Carlson A, Cheng H, Yu Q, Ahmed N, Wu J, Kim S, Sitti M, Ferreira PM, Huang Y, Rogers JA (2012). Adv. Mater..

[146] Zhou H, Qin W, Yu Q, Cheng H, Yu X, Wu H (2019). Nanomaterials.

[147] Lee CH, Kim DR, Zheng X (2014). ACS Nano.

[148] Yoon J, Jo S, Chun IS, Jung I, Kim H-S, Meitl M, Menard E, Li X, Coleman JJ, Paik U, Rogers JA (2010). Nature.

[149] Rogers JA, Lagally MG, Nuzzo RG (2011). Nature.

[150] Feng X, Qu B, Lu B, Zhao Z, Fang X (2011). Appl. Phys. Lett..

[151] Lee KJ, Meitl MA, Ahn J-H, Rogers JA, Nuzzo RG, Kumar V, Adesida I (2006). J. Appl. Phys..

[152] Sun Y, Menard E, Rogers JA, Kim HS, Kim S, Chen G, Adesida I, Dettmer R, Cortez R, Tewksbury A (2006). Appl. Phys. Lett..

[153] Seo J, Kim C, Ma BS, Lee T-I, Bong JH, Oh JG, Cho BJ, Kim T-S (2018). Adv. Funct. Mater..

[154] Abdelhalim A, Abdellah A, Scarpa G, Lugli P (2013). Carbon N. Y..

[155] Thanh QN, Jeong H, Kim J, Kevek JW, Ahn YH, Lee S, Minot ED, Park J-Y (2012). Adv. Mater..

[156] Pint CL, Xu Y-Q, Moghazy S, Cherukuri T, Alvarez NT, Haroz EH, Mahzooni S, Doorn SK, Kono J, Pasquali M, Hauge RH (2010). ACS Nano.

[157] J. Huang, F. Yeh, P. Lin, and C. Lu, in *2009 9th IEEE Conf. Nanotechnol.* (2009), pp. 674–677

[158] Ishikawa FN, Chang H, Ryu K, Chen P, Badmaev A, Gomez De Arco L, Shen G, Zhou C (2009). ACS Nano.

[159] Song D, Mahajan A, Secor EB, Hersam MC, Francis LF, Frisbie CD (2017). ACS Nano.

[160] Cao Q, Hur S-H, Zhu Z-T, Sun YG, Wang C-J, Meitl MA, Shim M, Rogers JA (2006). Adv. Mater..

[161] Kang SJ, Kocabas C, Kim H-S, Cao Q, Meitl MA, Khang D-Y, Rogers JA (2007). Nano Lett..

[162] Vilouras A, Heidari H, Gupta S, Dahiya R (2017). IEEE Trans. Electron Devices.

[163] Heidari H, Wacker N, Dahiya R (2017). Appl. Phys. Rev..

[164] Bedell SW, Fogel K, Lauro P, Shahrjerdi D, Ott JA, Sadana D (2013). J. Phys. D Appl. Phys..

[165] Yang J, Shen H, Jiang Y, Sun L (2019). Mater. Sci. Semicond. Process..

[166] Akinwande D, Petrone N, Hone J (2014). Nat. Commun..

[167] Cui Y, Lieber CM (2001). Science..

[168] Huang J-J, Liu C-J, Lin H-C, Tsai C-J, Chen Y-P, Hu G-R, Lee C-C (2008). J. Phys. D Appl. Phys..

[169] Takahashi T, Takei K, Adabi E, Fan Z, Niknejad AM, Javey A (2010). ACS Nano.

[170] Ahn J-H, Kim H-S, Lee KJ, Jeon S, Kang SJ, Sun Y, Nuzzo RG, Rogers JA (2006). Science..

[171] Liang X, Fu Z, Chou SY (2007). Nano Lett..

[172] Chang Y-K, Hong FC-N (2009). Nanotechnology.

[173] Gupta S, Yogeswaran N, Giacomozzi F, Lorenzelli L, Dahiya R (2020). Sens. J..

[174] Gupta S, Shakthivel D, Lorenzelli L, Dahiya R (2019). IEEE Sens. J..

[175] Gupta S, Giacomozzi F, Heidari H, Lorenzelli L, Dahiya R (2016). Procedia Eng..

[176] Yogeswaran N, Navaraj WT, Gupta S, Liu F, Vinciguerra V, Lorenzelli L, Dahiya R (2018). Appl. Phys. Lett..

[177] Heidari H, Bonizzoni E, Gatti U, Maloberti F, Dahiya R (2016). IEEE Sens. J..

[178] Gupta S, Heidari H, Vilouras A, Lorenzelli L, Dahiya R (2016). IEEE Trans. Circuits Syst. I Regul. Pap..

[179] C. G. Núñez, A. Vilouras, W. Taube Navaraj, F. Liu, and R. Dahiya, IEEE Sens. J. **18**, 7881 (2018)

[180] KimKim T, Lee SH, Li Y, Shi Y, Shin G, Lee SD, Huang Y, Rogers JA, Yu JS (2014). Appl. Phys. Lett.

[181] Yoon J, Baca AJ, Park S-I, Elvikis P, Geddes JB, Li L, Kim RH, Xiao J, Wang S, Kim T-H, Motala MJ, Ahn BY, Duoss EB, Lewis JA, Nuzzo RG, Ferreira PM, Huang Y, Rockett A, Rogers JA (2008). Nat. Mater..

[182] Service RF (2010). Science..

[183] Feng X, Yang BD, Liu Y, Wang Y, Dagdeviren C, Liu Z, Carlson A, Li J, Huang Y, Rogers JA (2011). ACS Nano.

[184] Zhu G, Yang R, Wang S, Wang ZL (2010). Nano Lett..

[185] Mai L, Gu Y, Han C, Hu B, Chen W, Zhang P, Xu L, Guo W, Dai Y (2009). Nano Lett..

[186] Yu G, Cao A, Lieber CM (2007). Nat. Nanotechnol..

[187] Bian R, Meng L, Zhang M, Chen L, Liu H (2019). ACS Omega.

[188] Zhou X, Zhou Y, Ku JC, Zhang C, Mirkin CA (2014). ACS Nano.

[189] Takei K, Takahashi T, Ho JC, Ko H, Gillies AG, Leu PW, Fearing RS, Javey A (2010). Nat. Mater..

[190] Cao Q, Kim HS, Pimparkar N, Kulkarni JP, Wang C, Shim M, Roy K, Alam MA, Rogers JA (2008). Nature.

[191] Lu W, Lieber CM (2007). Nat. Mater..

[192] Sevilla GAT, Hussain MM (2017). IEEE J. Emerg. Sel. Top. Circuits Syst..

[193] Rogers JA, Someya T, Huang Y (2010). Science..

[194] Martirosyan N, Kalani MYS (2011). Science..

[195] Dahiya RS, Cattin D, Adami A, Collini C, Barboni L, Valle M, Lorenzelli L, Oboe R, Metta G, Brunetti F (2011). IEEE Sens. J..

[196] Dahiya RS, Mittendorfer P, Valle M, Cheng G, Lumelsky VJ (2013). IEEE Sens. J..

[197] W. Dang, E. S. Hosseini, and R. Dahiya, in *2018 IEEE SENSORS* (2018), pp. 1–4

[198] Lipomi DJ, Vosgueritchian M, Tee BC-K, Hellstrom SL, Lee JA, Fox CH, Bao Z (2011). Nat. Nanotechnol..

[199] Lee G-H, Yu Y-J, Cui X, Petrone N, Lee C-H, Choi MS, Lee D-Y, Lee C, Yoo WJ, Watanabe K, Taniguchi T, Nuckolls C, Kim P, Hone J (2013). ACS Nano.

[200] Elías AL, Perea-López N, Castro-Beltrán A, Berkdemir A, Lv R, Feng S, Long AD, Hayashi T, Kim YA, Endo M, Gutiérrez HR, Pradhan NR, Balicas L, Mallouk TE, López-Urías F, Terrones H, Terrones M (2013). ACS Nano.

[201] Dahiya AS, Opoku C, Sporea RA, Sarvankumar B, Poulin-Vittrant G, Cayrel F, Camara N, Alquier D (2016). Sci. Rep..

[202] Dahiya AS, Opoku C, Oshman C, Poulin-Vittrant G, Cayrel F, Hue L-PTH, Alquier D, Camara N (2015). Appl. Phys. Lett..

[203] Liu F, Navaraj WT, Yogeswaran N, Gregory DH, Dahiya R (2019). ACS Nano.

[204] Liu Z, Liang B, Chen G, Yu G, Xie Z, Gao L, Chen D, Shen G (2013). J. Mater. Chem. C.

[205] Soni M, Dahiya R (2020). Philos. Trans. R. Soc. A Math. Phys. Eng. Sci.

[206] M. Soni, M. Bhattacharjee, M. Ntagios, and R. Dahiya, IEEE Sens. J. 1 (2020)

[207] Dang W, Manjakkal L, Navaraj WT, Lorenzelli L, Vinciguerra V, Dahiya R (2018). Biosens. Bioelectron..

[208] Lee J, Wu J, Shi M, Yoon J, Park S-I, Li M, Liu Z, Huang Y, Rogers JA (2011). Adv. Mater..

[209] Yoon J, Lee SM, Kang D, Meitl MA, Bower CA, Rogers JA (2015). Adv. Opt. Mater..

[210] Dang W, Vinciguerra V, Lorenzelli L, Dahiya R (2017). Flex. Print. Electron..

[211] Dahiya AS, Thireau J, Boudaden J, Lal S, Gulzar U, Zhang Y, Gil T, Azemard N, Ramm P, Kiessling T, O’Murchu C, Sebelius F, Tilly J, Glynn C, Geary S, O’Dwyer C, Razeeb KM, Lacampagne A, Charlot B, Todri-Sanial A (2020). J. Electrochem. Soc..

[212] Dahiya RS, Metta G, Valle M, Sandini G (2010). IEEE Trans. Robot..

[213] Dahiya RS, Gori M (2010). J. Neurophysiol..

[214] Kim T-S, Lee Y, Xu W, Kim YH, Kim M, Min S-Y, Kim TH, Jang HW, Lee T-W (2019). Nano Energy.

[215] Lee HE, Park JH, Kim TJ, Im D, Shin JH, Kim DH, Mohammad B, Kang IS, Lee KJ (2018). Adv. Funct. Mater..

[216] Jeong JW, Yang SR, Hur YH, Kim SW, Baek KM, Yim S, Jang H-I, Park JH, Lee SY, Park C-O, Jung YS (2014). Nat. Commun..

[217] M. Ntagios, H. Nassar, A. Pullanchiyodan, W. T. Navaraj, and R. Dahiya, Adv. Intell. Syst. 1900080 (2020)

[218] Ota H, Emaminejad S, Gao Y, Zhao A, Wu E, Challa S, Chen K, Fahad HM, Jha AK, Kiriya D, Gao W, Shiraki H, Morioka K, Ferguson AR, Healy KE, Davis RW, Javey A (2016). Adv. Mater. Technol..

[219] M. Bhattacharjee, M. Soni, P. Escobedo, and R. Dahiya, Adv. Electron. Mater. (2020)M. Bhattacharjee, M. Soni, P. Escobedo, and R. Dahiya, Adv. Electron. Mater. (2020)

[220] Jamshidi A, Pauzauskie PJ, Schuck PJ, Ohta AT, Chiou P-Y, Chou J, Yang P, Wu MC (2008). Nat. Photonics.

[221] Shin JC, Mohseni PK, Yu KJ, Tomasulo S, Montgomery KH, Lee ML, Rogers JA, Li X (2012). ACS Nano.

[222] Shin JC, Lee A, Mohseni PK, Kim DY, Yu L, Kim JH, Kim HJ, Choi WJ, Wasserman D, Choi KJ, Li X (2013). ACS Nano.

